# *MALAT1-*KTN1-EGFR regulatory axis promotes the development of cutaneous squamous cell carcinoma

**DOI:** 10.1038/s41418-019-0288-7

**Published:** 2019-01-25

**Authors:** Ying Zhang, Lin Gao, Shudong Ma, Ji Ma, Yinghui Wang, Shanshan Li, Xia Hu, Shuo Han, Meijuan Zhou, Liang Zhou, Zhenhua Ding

**Affiliations:** 1 Department of Radiation Medicine, Guangdong Provincial Key Laboratory of Tropical Disease Research, School of Public Health, Southern Medical University, Guangzhou, 510515 China; 2 Department of Oncology, Nanfang Hospital, Southern Medical University, Guangzhou, 510515 China

**Keywords:** Oncogenes, Oncogenes

## Abstract

Long noncoding RNAs (LncRNAs), including *MALAT1*, are critical regulators of tumor development. However, the roles and molecular mechanisms of LncRNAs in cutaneous squamous cell carcinoma (cSCC) remain underexplored. In this study, functional studies using in vitro cellular and in vivo xenograft models confirmed the pro-carcinogenic roles of *MALAT*1 in cSCC. Further, *MALAT1* was identified to regulate epidermal growth factor receptor (EGFR) protein expression but did not affect *EGFR* mRNA expression. Transcriptomic sequencing identified kinectin 1 (KTN1) as the key mediator for *MALAT1* regulation of EGFR. Mechanistic study revealed that *MALAT1* interacts with c-MYC to form a complex and directly binds to the promoter region of *KTN1* gene and enhances its transactivation to positively regulate EGFR protein expression. Our findings, therefore, establish a novel c-MYC-assisted *MALAT1*-KTN1-EGFR axis, which contributes to cSCC development and may serve as novel target for therapeutic intervention.

## Introduction

Cutaneous squamous cell carcinoma (cSCC) represents the second most common cancer worldwide with an annual accidence over one million individuals [1, 2]. cSCC most frequently develops in skin that receives chronic sun exposure and generates ultraviolet (UV)-induced DNA damage in epidermal keratinocytes, leading to the development cSCC and metastases may eventually develop [1, 3]. However, the underlying molecular mechanism responsible for the development of cSCC remains obscure.

Recently, LncRNAs longer than 200 nucleotides have been shown to play critical roles in pathophysiological processes, e.g., neurogenesis, myogenesis, and carcinogenesis [4]. LncRNAs can act as a scaffold to tether and coordinate different factors for specific regulatory functions like HOTAIR or as a guide for recruiting transcription factors and their co-factors to specific sites of chromatin to regulate the expression of downstream genes and further influence the malignant hallmarks of cancer like LincRNA-p21 [5, 6].

*Metastasis-associated lung adenocarcinoma transcript 1* (*MALAT1*), a 8000 nt-long single-exon noncoding transcript highly expressed in nucleus, is upregulated in many solid cancers and regulates alternative splicing or transcription to promote cancer progression, metastasis, and recurrence [7–9]. In lung cancer, the migration and metastasis capacity of tumor cells was markedly compromised in *MALAT1*-deficient cells owing to the decreased expression of proliferation, invasiveness, and metastasis-related genes [10]. Notably, *MALAT1* functions as a scaffold by associating with histone modifiers such as LSD1, SET2, and MLL to regulate E2F-dependent target gene expression [11] and also serves as a ‘‘nuclear hub’’ for storage and/or sequestration of RNA-binding proteins [9]. However, the roles of *MALAT1* in cSCC still remains poorly understood.

In this study, *MALAT1* was characterized to be highly expressed in cSCC tumors and cell lines. Depletion of *MALAT1* repressed the proliferation, migration, and invasiveness but promotes apoptosis in cSCC. Further investigation revealed that significantly upregulated EGFR protein is modulated by *MALAT1* in cSCC. Transcriptomic analysis identified kinectin 1 (KTN1) as the key mediator for *MALAT1* regulation of EGFR. *MALAT1* physically interacts with c-MYC, promotes its chromatin recruitment, and binds directly to the *KTN1* promoter region to transactivate *KTN1* expression for enhancing EGFR protein expression. In vivo and in vitro identification of this novel *MALAT1*-KTN1-EGFR axis deepens our understanding of the pivotal roles of LncRNAs in cSCC progression and also provides new target for anti-cancer intervention.

## Results

### *MALAT1* is induced by UVB irradiation and highly expressed in cSCC cells and tumors

**Fig. 1:**
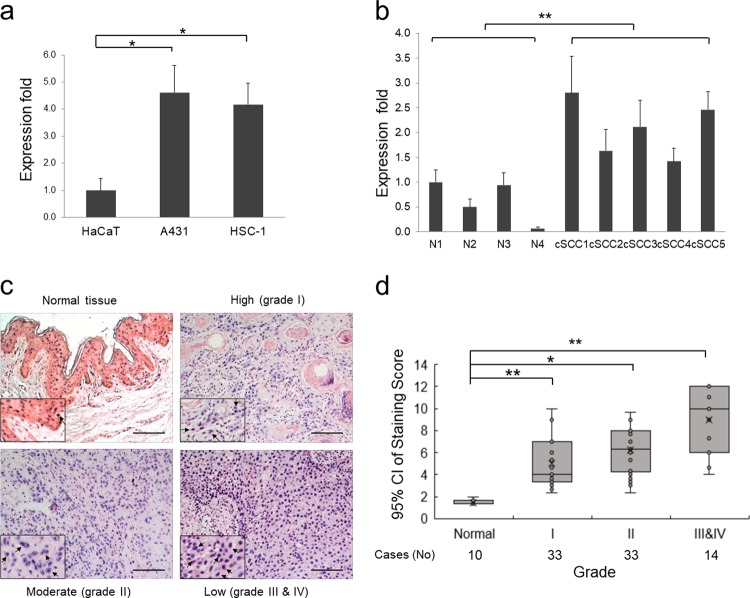
*MALAT1* is induced by UV irradiation and is overexpressed in cSCC cell lines and tumors. **a** Expression levels of *MALAT1* were detected in cSCC cell lines and control HaCaT keratinocytes by qRT-PCR. **b** Expression levels of *MALAT1* were detected in the normal tissues and cSCC tumors by qRT-PCR. **c** RNA ISH staining of *MALAT1* on cSCC specimens. *MALAT1* staining images representing normal tissue and low, medium, and high cSCC differentiation grades are shown. Positive *MALAT1* signals are NBT/BCIP precipitates in a purple blue color and indicated by arrow heads. **d** Association of *MALAT1* ISH-staining scores with grades of cSCC tumor differentiation (low, medium, or high). Case numbers are indicated below. Data are plotted as the means of the 95% confidence intervals. All statistical data represent the average of three independent experiments ± s.d. *P *<* 0.05, **P *<* 0.01, ***P *<* 0.001. Scale bars, 100 µm

To screen LncRNAs potentially functioning in cSCC, we collected diseased-related LncRNAs list from LncRNADisease database (http://cmbi.bjmu.edu.cn/lncrnadisease) [12], analyzed with GenClip 2.0 (http://ci.smu.edu.cn/GenCLiP2/) [13] and obtained ten most-studied LncRNAs (Supplementary Fig. S1a). Quantitative reverse transcription PCR (qRT-PCR) detection indicated that *MALAT1* is stably and markedly higher expressed in all cSCC cell lines with similar folds compared with other LncRNAs, which may indicate its tightly relationship with cSCC development (Fig. 1a and Supplementary Fig. S1b–j). To investigate the relationship between UV exposure and *MALAT1* expression, HaCaT keratinocytes were treated with ultraviolet B (UVB) and checked for the expression of *MALAT1*. As predicted, *MALAT1* was continuously induced by UVB treatment (Supplementary Fig. S2a). Interestingly, this continuous induction profiles of *MALAT1* were also detected in both of the A431 and HSC-1 cSCC cell lines after UVB exposure (Supplementary Fig. S2b, c).

Consistent with the cellular results, *MALAT1* exhibited much higher expression in cSCC primary tumors compared to normal tissues (Fig. 1b). Further, *MALAT1* expression was also examined in paraffin-embedded sections of 80 cSCC and ten normal specimens by in situ hybridization (ISH). Nuclear-localized positive signals of *MALAT1* at various levels (weak, moderate, or strong) were shown in all tumors examined, whereas all of the normal skin specimens showed weak *MALAT1* signals (Fig. 1c). Scoring of *MALAT1* staining revealed that it correlates positively with the ascending cSCC grades. Specifically, an evident increasing trend was observed across normal tissue and the early grades of cSCC (grade I and II) (*P* < 0.05), and also extended to late stage cSCC (III and IV) (*P* < 0.05) (Fig. 1d). Collectively, the above results suggest that UV-inducible *MALAT1* is upregulated in cSCC cells and primary tumors.

### *MALAT1* promotes proliferation, migration, and invasiveness but represses apoptosis of cSCC cells

**Fig. 2:**
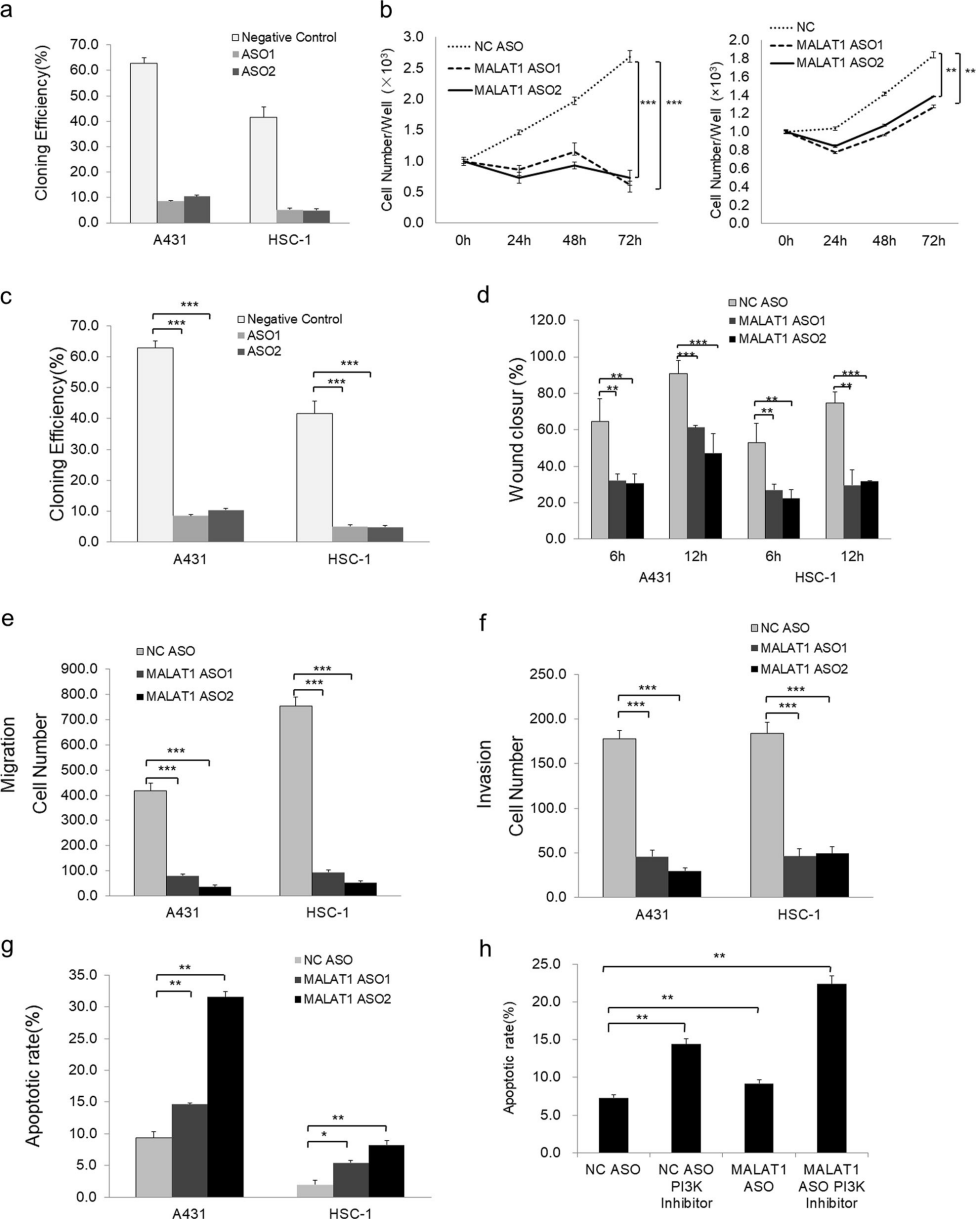
Knockdown of *MALAT1* inhibits cell proliferation, mobility, migration, and invasion but promotes apoptosis of both A431 and HSC-1 cells. **a** Knockdown of *MALAT1* in A431 and HSC-1 cells by *MALAT1* ASO1 and *MALAT1* ASO2 was determined by qRT-PCR assay. **b** CCK-8 assay determination of A431 and HSC-1 cell proliferation in response to *MALAT1* knockdown. **c** Monolayer colony formation assay showing that *MALAT1* knockdown significantly decreased the colony formation capacity of A431 and HSC-1 cells. The colony numbers were counted and recorded. **d** Wound-healing assay showing that knockdown of *MALAT1* significantly decreased the wound closure ability of A431 and HSC-1 cells at both 6 and 12 h. **e** Transwell assay indicated that *MALAT1* knockdown suppressed A431 and HSC-1 cell motility. **f** Matrigel invasion assay indicated that *MALAT1* knockdown drastically inhibited A431 and HSC-1 cell invasiveness. **g** Flow cytometric analysis using Annexin V/PI staining showing that *MALAT1* silencing significantly increased the apoptosis of A431 and HSC-1 cells (*P *<* 0.05, **P *<* 0.01 with LSD test of one-way ANOVA). **h** PI3K inhibitor LY294002 treatment led to much high apoptotic rate in addition to *MALAT1* depletion-induced apoptosis. All statistical data represent the average of three independent experiments ± s.d. *P *<* 0.05, **P *<* 0.01, ***P *<* 0.001

The upregulation of *MALAT1* in cSCCs implied that it may play an oncogenic role in cSCC development. To test this notion, significant knockdown of *MALAT1* RNA was achieved using antisense oligonucleotides (ASOs), which led to the significant decreases of cell proliferation and colony formation capacity were detected after *MALAT1* knockdown in both A431 and HSC-1 cell lines (Fig. 2a–c and Supplementary Fig. S3a). Furthermore, much slower wound closure of the monolayer in wound-healing assay, fewer cells penetrating of the membrane pores in transwell assay and fewer cells penetrating the gel layer in Matrigel invasiveness measurement after *MALAT1* knockdown indicated the significant inhibition of migration and invasiveness abilities in both A431 and HSC-1 cSCC cells (Fig. 2d–f and Supplementary Fig. S3b–d). To further explore the roles of *MALAT1* in cSCC, we did gain-of-function validations and confirmed that *MALAT1* promotes proliferation, migration, and invasiveness in both A431 and HSC-1 cells (Supplementary Figs. S4a–f and S5a–f).

To assess the effect of *MALAT1* on apoptosis of cSCC cells, Annexin V/propidium iodide (PI) double staining revealed that knockdown of *MALAT1* induced a reduction in the living cell population along with an accompanying increase in the apoptotic population, while overexpression of *MALAT1* significantly repressed apoptosis in both A431 and HSC-1 cells (Fig. 2g and Supplementary Figs. S3e, S4g, and S5g). To explore the potential mechanisms leading to apoptosis by *MALAT1* knockdown, we detected the variations of master factors in STAT3, PI3K, AKT, and mTOR signaling pathways. From the results, the PI3K pathway was significantly influenced in both overexpression and knockdown of *MALAT1* conditions (Supplementary Fig. S6a, b). Consistently, PI3K inhibitor LY294002 treatment led to much higher apoptotic rate in addition to *MALAT1* depletion-induced apoptosis (Fig. 2h and Supplementary Fig. S6c). Altogether, the above data demonstrated that *MALAT1* plays pro-carcinogenic roles in cSCC.

### EGFR protein is regulated by *MALAT1*

**Fig. 3:**
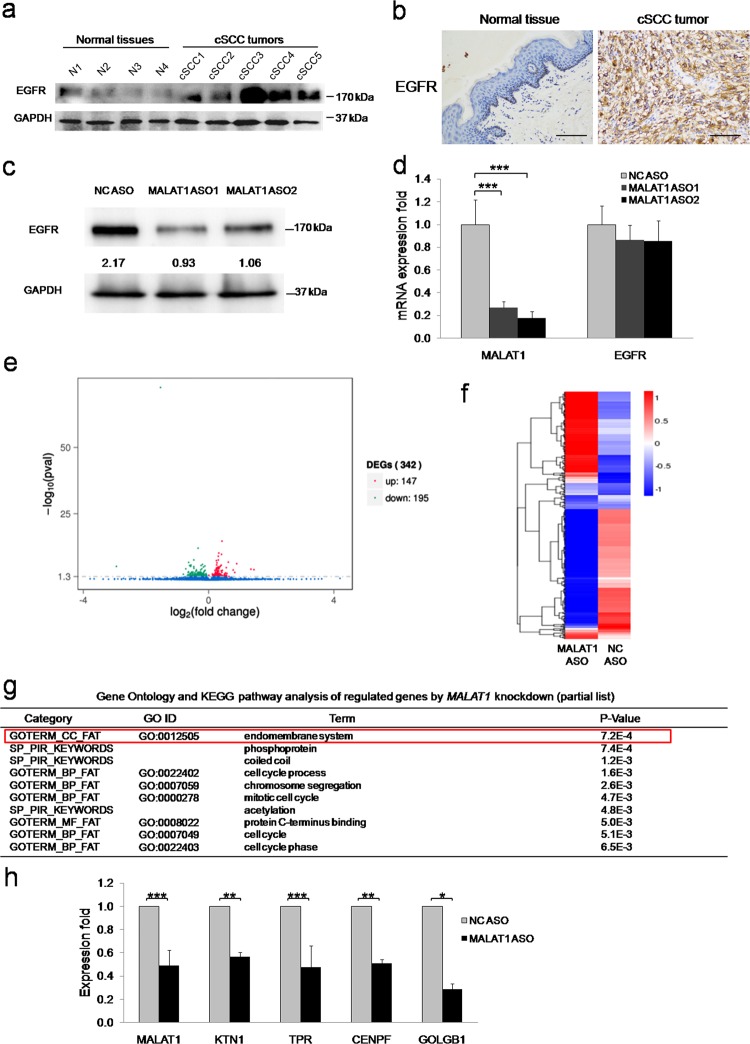
Genome-wide analysis by RNA-Seq of transcriptome changes consequent to *MALAT1* knockdown to identify the mediator for *MALAT1* regulation of EGFR protein expression. **a** EGFR was detected in normal tissues and cSCC tumors by western blot, which indicated that EGFR are overexpressed in cSCC tumors. **b** EGFR was detected in normal tissues and cSCC tumors by IHC. Representative images confirmed that EGFR is overexpressed in cSCC tumors. **c** Western blot analysis indicated that EGFR protein expression was significantly downregulated upon *MALAT1* knockdown. **d** qRT-PCR showing that depletion of *MALAT1* did not affect the mRNA expression of *EGFR*. Total RNAs were analyzed by qRT-PCR for *MALAT1* and *EGFR* expression. **e** Total RNAs were isolated from A431 cells treated with NC ASO or *MALAT1* ASO oligos and subjected to high throughput mRNA sequencing. Differentially expressed genes are shown by volcano plot. **f** Differentially expressed genes were clustered and shown in a heat map. Color bars at the right represent gene clusters established through k-means clustering. The cluster heat map uses the FPKM as the input value for hierarchical clustering analysis. The log10(rpkm + 1) is input into the pheatmap module of the R package. The software normalizes the FPKM values of all samples. The maximum value is normalized to 1, and the minimum value is normalized to –1 to indicate the expression profiles of differential genes or differential transcripts under different experimental conditions. **g** Over-represented Gene Ontology categories by Gene Ontology analysis of differently expressed genes. BP: biological process; MF: molecular function; CC: cellular component; KEGG: Kyoto Encyclopedia of Genes and Genomes. “Endomembrane system” is highlighted. **h** Validation of *MALAT1* and four downregulated common genes in ‘‘endomembrane system’’, ‘‘phosphoprotein’’, and ‘‘coiled coil’’ categories by qRT-PCR. All statistical data represent the average of three independent experiments ± s.d., while the standard error of the mean (s.e.) was used in **h**. *P *<* 0.05, **P *<* 0.01, ***P *<* 0.001. Scale bars, 100 µm

As the A431 cell line is featured in *EGFR* gene amplification [14], the strong tumor-suppressive effect by *MALAT1* depletion suggested that EGFR might be regulated by *MALAT1*. To test this hypothesis, IHC and western blot assays were firstly performed and revealed that EGFR protein is higher expressed in cSCC tumors than in normal skin tissues (Fig. 3a, b), which coordinated with *MALAT1* expression in cSCC (Fig. 1d). Importantly, loss of *MALAT1* led to the significant downregulation of EGFR protein expression, which clearly indicated that *MALAT1* may directly or indirectly regulate EGFR expression (Fig. 3c). In contrast, qPCR detection was unable to confirm a significant downregulation of *EGFR* mRNA expression upon *MALAT1* knockdown (Fig. 3d), which is consistent with previous studies [10, 15]. Based on these results, we concluded that nucleus-localized *MALAT1* might regulate the protein expression but not the mRNA expression of *EGFR*.

### *MALAT1* mediates transcriptomic changes in cSCC cells

To characterize *MALAT1*-mediated transcriptomic changes in cSCC cells, transcriptomic sequencing (RNA-Seq) was conducted after *MALAT1* knockdown. The quality of RNA-Seq was shown by more than 90% of the reads mapped to exonic regions, which excluded the contamination of genomic DNA and unspliced pre-mRNA and indicated high specificity of *MALAT1* effects on expressed mRNA (Supplementary Fig. S7). Initial analysis of differentially expressed genes with *P*-value < 0.05 identified that 147 genes were upregulated, whereas 195 were downregulated (Fig. 3e, f and Supplementary Table 1). Further analysis with more stringent *q*-value ( < 0.05) yielded a total of 23-upregulated and 16-downregulated genes, respectively (Supplementary Table 2). The 16-downregulated gene list was subsequently submitted to the DAVID Bioinformatics Resources (https://david.ncifcrf.gov) for Gene Ontology (GO) analysis. The functional annotation chart revealed that ‘‘endomembrane system’’, ‘‘phosphoprotein’’, and ‘‘coiled coil’’ were ranked as the top three GO categories, which shared four common genes: *CENPF*, *KTN1*, *TPR*, and *GOLGB1* (Fig. 3g and Supplementary Table 3). These four genes were consistently downregulated in subsequent qRT-PCR validation experiments in concordance with *MALAT1* knockdown (Fig. 3h). Specially, KTN1 is functionally associated with protein synthesis by association with the EEF-1 complex [16], indicating KTN1 may participate in the regulation of EGFR protein expression by *MALAT1*. Importantly, the specific downregulation of KTN1 upon *MALAT1* knockdown was also verified by another RNA-seq analysis in HSC-1 cSCC cell line, while the *EGFR* RNA expression is still not influenced (Supplementary Fig. S8). These observations suggested that *MALAT1* potentially targets KTN1 to regulate EGFR protein expression.

### KTN1 represents the key mediator for *MALAT1*-regulated EGFR protein expression and tumor progression in cSCC

**Fig. 4:**
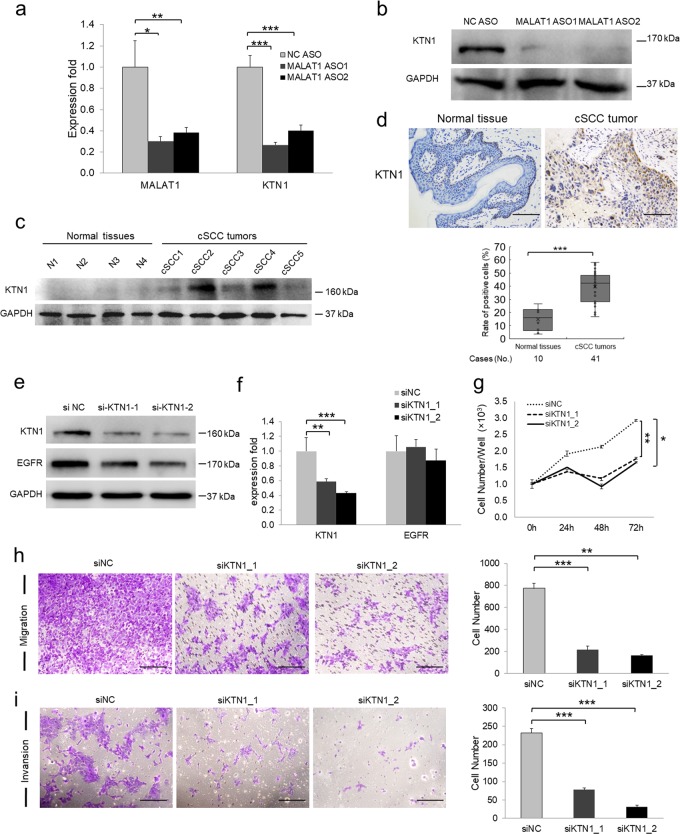
KTN1 mediates *MALAT1* regulation of EGFR in cSCC. **a** RNA expression of *MALAT1* and *KTN1* was examined in A431 cells with two different *MALAT1* ASOs by qRT-PCR. **b** KTN1 protein expression was examined in *MALAT1* depleted A431 cells by western blot, which showed significant loss of KTN1 in response to *MALAT1* knockdown. **c** KTN1 was detected in normal tissues and cSCC tumors by western blot. **d** KTN1 expression was detected in normal tissues and cSCC tumors by IHC. Representative images confirmed that KTN1 is overexpressed in cSCC tumors. **e** RNA interference (RNAi) with siRNAs targeting KTN1 was applied to A431 cells and the protein expression of KTN1 and EGFR was detected using western blot. **f** RNA expression of *KTN1* and *EGFR* was analyzed by qRT-PCR to determine the efficiency and specificity of *KTN1* RNA knockdown. **g** CCK-8 assay determination of A431 cell proliferation in response to KTN1 knockdown (*P *=* 0.0480 with Dunnett T3 test of one-way ANOVA). **h** Transwell assay of A431 cell motility following KTN1 knockdown. **i** Matrigel invasion assay of A431 cell invasiveness following KTN1 knockdown. All statistical data represent the average of three independent experiments ± s.d. *P *<* 0.05, **P *<* 0.01, ***P *<* 0.001. Scale bars, 100 µm

KTN1 was significantly downregulated upon *MALAT1* knockdown at both RNA and protein levels (Fig. 4a, b). Also, western blot and IHC assays revealed that KTN1 were highly expressed in cSCC tumors (Fig. 4c, d), which indicates a potential relationship of EGFR and KTN1 together with highly expressed *MALAT1* in cSCC tumors (Fig. 1d). To explore whether KTN1 serves as the mediator for *MALAT1* regulation of EGFR, RNA interference of KTN1 using siRNA oligos was performed and a marked loss of KTN1 protein was detected in siKTN1-transfected cells compared with NC treatment (siNC) (Fig. 4e). As expected, EGFR protein expression levels were downregulated together following KTN1 depletion. However, knockdown of *KTN1* had no effect on the RNA expression of *EGFR* (Fig. 4f). Notably, knockdown of KTN1 inhibited cell proliferation, migration, and invasion, which is consistent with the effects of *MALAT1* depletion (Fig. 4g–i). The *MALAT1*-KTN1-EGFR regulatory axis and the pro-carcinogenic function of KTN1 were also validated in another cSCC cell line HSC-1 (Supplementary Fig. S9a–g).

To explore if the above finding is widely existed in different types of cancers, we also knocked down *MALAT1* and then detected KTN1 and EGFR expression in cervical cancer cell line Hela and melanoma cell line A375. As expected, depletion of *MALAT1* significantly downregulated KTN1 and EGFR protein expression, whereas *KTN1* mRNA was also knocked down but no change was observed in *EGFR* mRNA expression (Supplementary Fig. S10a, b).

To further confirm the regulatory mechanism, we performed gain-of-function study. As expected, EGFR protein expression levels were significantly upregulated following *MALAT1* overexpression. Yet, overexpression of *MALAT1* still had no effect on the *EGFR* mRNA expression while *KTN1* mRNA level was significantly increased in both A431 and HSC-1 cSCC cell lines (Supplementary Figs. S11a, b and  S12a, b).

Altogether, the above results indicated that KTN1 regulates EGFR at the protein level but not at the RNA level, which is consistent with the role of *MALAT1* and clearly indicated that KTN1 serves as the mediator of *MALAT1* function in regulating EGFR protein expression.

### *MALAT1* directly interacts with c-MYC and binds to the promoter region to transactivate *KTN1*

**Fig. 5:**
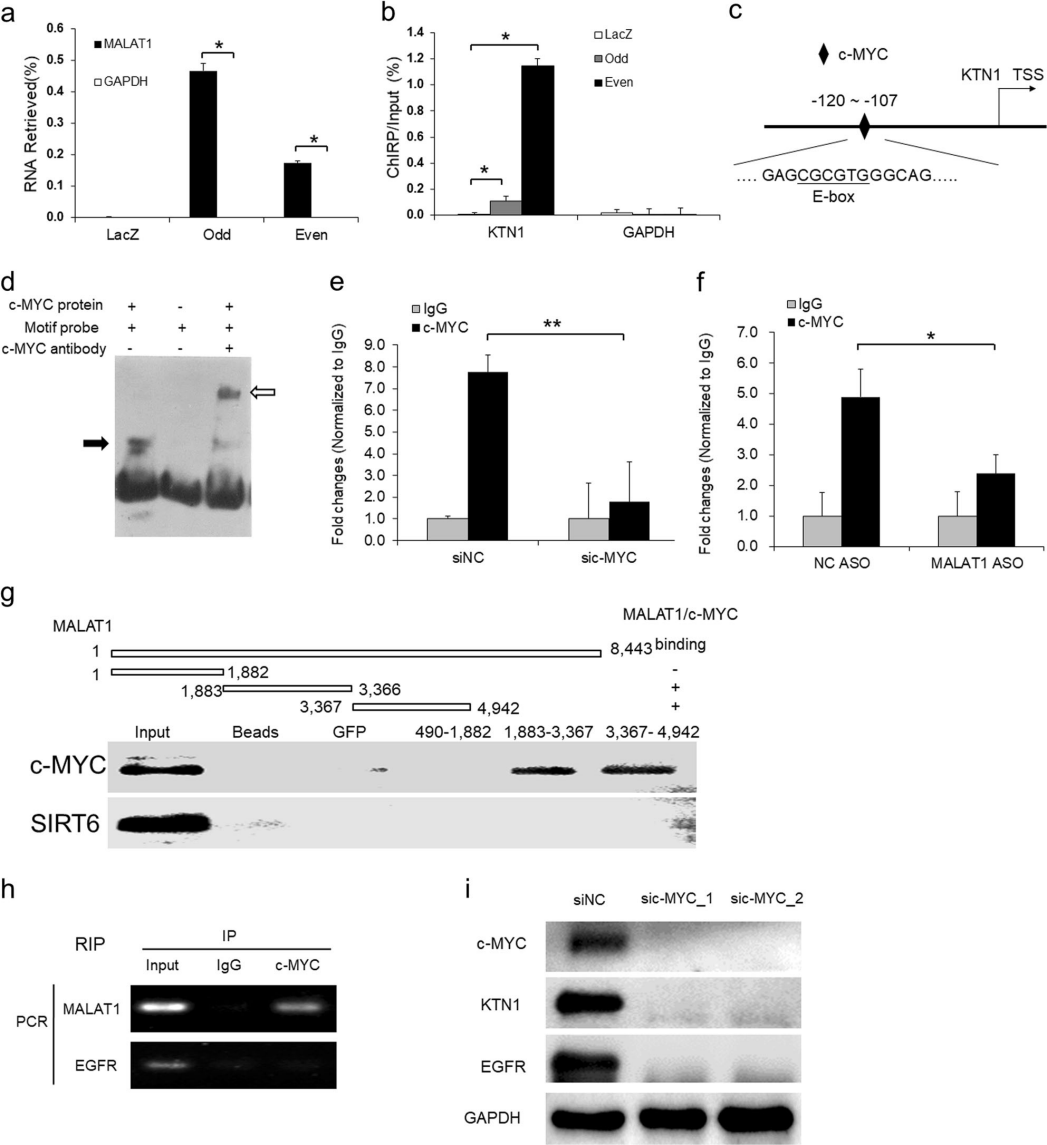
*MALAT1* directly interacts with c-MYC for binding to the promoter region of *KTN1* gene to mediate the transcriptional regulation of *KTN1* expression. **a** Chromatin isolation by RNA purification (ChIRP) with both even and odd antisense oligos tiling *MALAT1* showing degree and specificity of *MALAT1* retrieval. LacZ tiling oligo retrieval of *MALAT1* RNA was used as a negative control. **b** Relative retrieval of genomic DNAs corresponding to the *KTN1* promoter or *GAPDH* locus following *MALAT1* or LacZ ChIRP. **c** Identification of the E-box element for c-MYC binding at the *KTN1* promoter region. TSS, transcription start site. **d** Electrophoretic mobility shift assay (EMSA) was used to detect the direct association between the E-box element and purified c-MYC protein. Black arrow indicates the binding complex between c-MYC protein and probe containing E-box motif while the white arrow indicates the supershift generated by the association of anti-c-MYC antibody with the above complex. **e** c-MYC siRNA and NC oligos were transfected into A431 cells. The enrichment of c-MYC at the *KTN1* promoter was measured by ChIP-PCR. **f** *MALAT1* and NC ASOs were transfected into A431 cells. The enrichment of c-MYC at the *KTN1* promoter was measured by ChIP-qPCR. **g** Biotin-labeled fragment 1, fragment 2, and fragment 3 domains of *MALAT1* RNA transcripts were used to retrieve c-MYC protein by RNA pull-down assay. Random selected protein, SIRT6, was used as a negative control. **h** RNA immunoprecipitation (RIP) assay was performed using antibodies against c-MYC. The retrieved *MALAT1* transcripts were detected by RT-PCR. *EGFR* transcripts were used as a negative control. **i** RNA interference (RNAi) with siRNAs targeting c-MYC was applied to A431 cells and the protein expression of c-MYC, KTN1, and EGFR was detected using western blot. All statistical data represent the average of three independent experiments ± s.d. *P *<* 0.05, **P *<* 0.01, ***P *<* 0.001

To explore the mechanism of *MALAT1* regulation of KTN1, ChIRP assay with both odd and even tiling oligos against *MALAT1* was performed. The results demonstrated that endogenous *MALAT1* RNA but not *glyceraldehyde-3-phosphate dehydrogenase* (*GAPDH*) RNA was retrieved from chromatin, whereas negative control LacZ tiling oligos retrieved no *MALAT1* RNA (Fig. 5a). At the same time, a significant amount of genomic DNA corresponding to the *KTN1* promoter region was obtained whereas no *GAPDH* locus sequences were retrieved; in comparison, LacZ ChIRP retrieved no signal in either locus (Fig. 5b). We also validated the specific binding of *MALAT1* to the *KTN1* promoter using HSC-1 cell lines and obtained similar results (Supplementary Fig. S13a–d). These results indicated that *MALAT1* physically interacted with the promoter region of *KTN1*, suggesting the occurrence of potentially direct regulation at the transcriptional level.

To investigate the interacting protein partner(s) of *MALAT1* at the target loci, we interrogated the *KTN1* promoter region in UCSC genome browser (https://genome.ucsc.edu/) for candidate. Among the transcription factors binding to *KTN1* promoter region, c-MYC exhibits relatively strong binding across the transcriptional start site of *KTN1* as does its dimerization partner, MYC-associated protein X (MAX) (Supplementary Fig. S14). Notably, the core motif for c-MYC-MAX heterodimer binding, the E-box sequence (CGCGTG), was also identified within the predicted c-MYC binding region (Fig. 5c) using the rVista 2.0 web tool [17]. The physical binding of c-MYC at the E-box site was confirmed by electrophoretic mobility shift assay (EMSA) (Fig. 5d). Subsequent result from chromatin immunoprecipitation (ChIP)-qPCR clearly confirmed that c-MYC directly binds at the *KTN1* promoter region and this enrichment is specifically decreased in response to the depletion of c-MYC (Fig. 5e). Further, to verify whether *MALAT1* is required for the binding of c-MYC at *KTN1* promoter, ChIP assay was performed and showed that c-MYC binding enrichment decreased upon *MALAT1* depletion (Fig. 5f). Importantly, the above molecular mechanism was also verified in another cSCC cell line HSC-1 (Supplementary Fig. S13a–g). Thus, the binding of c-MYC at *KTN1* promoter is dependent on *MALAT1*.

To investigate whether *MALAT1* directly interacts with c-MYC, an RNA pull-down assay was performed from native non-cross-linked cell lysates using in vitro-synthesized and biotinylated RNA fragments of *MALAT1*. Western blot analysis confirmed that c-MYC could directly bind to fragments 2 and 3 of *MALAT1*, whereas the bead control, GFP, and fragment 1 showed no signal (Fig. 5g). The interaction between *MALAT1* and c-MYC was further strengthened by the results of RNA immunoprecipitation (RIP) using a c-MYC antibody (Fig. 5h). To verify whether c-MYC is required for the regulation of KTN1 by *MALAT1*, depletion of c-MYC was performed and led to drastic loss of KTN1 and EGFR protein expression in both A431 (Fig. 5i) and HSC-1 (Supplementary Fig. S13h). Collectively, our findings demonstrate that c-MYC is critical for *MALAT1* regulation of KTN1 and the further downstream regulatory control of EGFR.

### In vivo function of the MALAT1-KTN1-EGFR regulatory axis

**Fig. 6:**
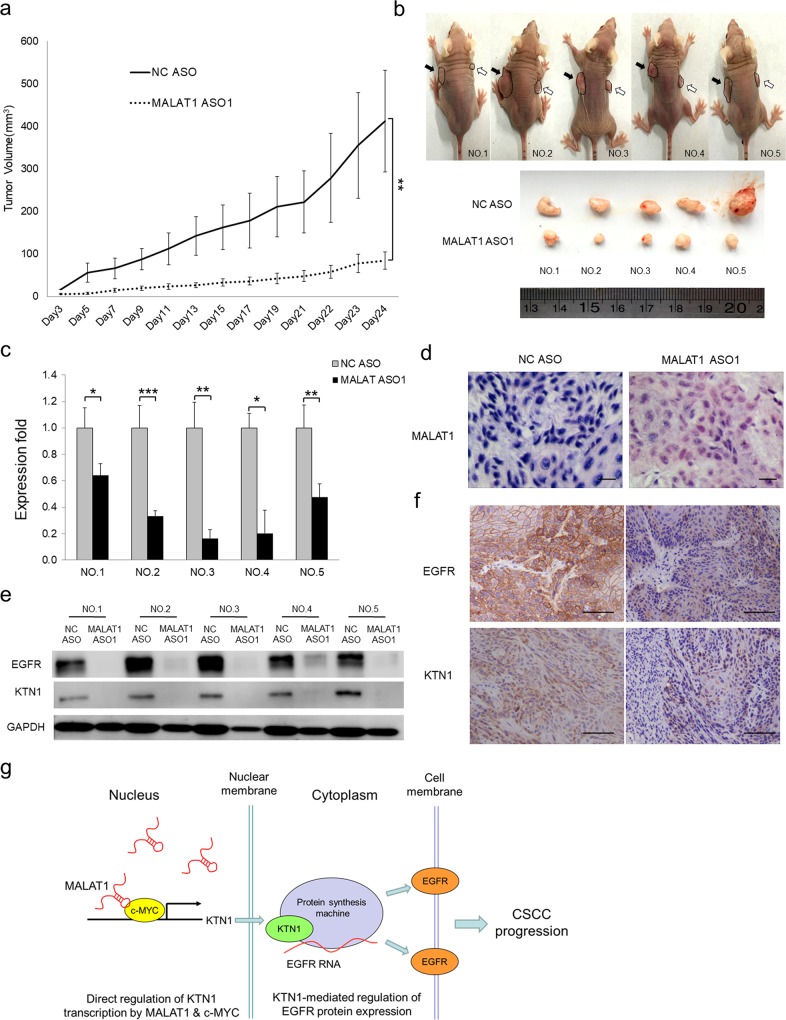
The *MALAT1*-KTN1-EGFR regulatory axis exists in vivo. *MALAT1* ASO were injected into A431 cell xenografts every 2 days. **a** Loss of *MALAT1* attenuates subcutaneous tumor growth in a mouse xenograft model. Tumor volumes (mm^3^) were plotted according to days. Tumor volumes statistical data represent the average of five independent experiments ± s.d, respectively. **b** The mice were sacrificed at the end of the experiment and images taken along with the dissected tumors from five representative mice are shown. Black arrows indicate NC ASO-treated xenografts while white arrows indicate *MALAT1* ASO-treated xenografts. **c** The expression of *MALAT1* in the dissected tumors was measured by qRT-PCR. qRT-PCR statistical data represent the average of three independent experiments ± s.d, respectively. **d** The protein expression of KTN1 and EGFR was detected in the xenografts. **e** RNA in situ hybridization assay was used to detect expression of *MALAT1* in NC and *MALAT1* ASO-treated xenografts. Scale bar, 50 µm. **f** Histopathology analysis (IHC staining) showing the marked loss of the expression of EGFR and KTN1 in xenografts. Scale bar, 100 µm. *P *<* 0.05, **P *<* 0.01, ***P *<* 0.001. **g** The model depicts the roles of the *MALAT1*-KTN1-EGFR regulatory axis. In cSCC tumors, the aberrantly expressed *MALAT1* interacts with c-MYC to form a complex, binds to the promoter region of *KTN1* gene and transcriptionally enhances KTN1 expression. The upregulated KTN1 may associate with protein translation machine to promote EGFR expression at the protein level, which contributes to cSCC development

To validate the pro-carcinogenic role of *MALAT1*-KTN1-EGFR axis in vivo, loss of *MALAT1* was achieved by injecting *MALAT1* ASO into xenograft tumors established in immunocompromised mice. The injections were repeated every other day for 24 days. Significant differences in tumor volumes could be observed after 5 days and persisted until the experimental endpoint (Fig. 6a), at which point NC ASO injected tumors were much larger than those injected with *MALAT1* ASO (*n* = 5; *P* = 0.00156) (Fig. 6b). Tumors were harvested and resected for further evaluation. qRT-PCR and ISH staining revealed that *MALAT1* ASO oligo injection effectively led to significant losses of *MALAT1* expression (Fig. 6c, d). Western blot and IHC staining indicated that the protein expression of *MALAT1* downstream targets, including KTN1 and EGFR, was drastically downregulated in response to *MALAT1* depletion (Fig. 6e, f). The existence of *MALAT1*-KTN1-EGFR regulatory axis was also verified in vivo using another *MALAT* ASO (Supplementary Fig. S16). Importantly, *MALAT1* overexpression promoted tumor growth when compared with the vector control (Supplementary Fig. S17a). Tumor size and mass were evidently smaller at the end of evaluation (Supplementary Fig. S17b). *MALAT1* overexpression, as shown by qRT-PCR and ISH staining (Supplementary Fig. S17c, e), significantly enhanced the expression of KTN1 and EGFR proteins as shown by western blot and IHC staining (Supplementary Fig. S17d, f).

## Discussion

The functional and pathophysiological relevance of LncRNAs have been proposed by increasing evidence linking the dysregulation of LncRNAs with diverse human diseases from diabetes complications to cancers by regulating alternative splicing, nuclear organization, and epigenetic modulation of gene expression [9, 18]. Yet, the role of *MALAT1* in the tumorigenesis and pathogenesis of cSCC remains underexplored.

Serving as an activator of the MAPK, PI3K, and KRAS pathways, EGFR contributes to proliferation and metastasis of cancer cells and can be activated upon UV exposure [19]. Blockade of EGFR function inhibits the subsequent activation of EGFR downstream signaling cascades, which makes EGFR to be an optimal target for cancer therapy including cSCC [20]. Considering the importance of EGFR in cSCC development, the regulatory mechanisms controlling EGFR protein expression is still unclear in cSCC. In this study, our results showed that the loss of pro-carcinogenic *MALAT1* led to the inhibition of EGFR protein expression but does not affect *EGFR* mRNA expression.

LncRNAs have been shown to regulate protein synthesis or stability. LncMyoD directly binds to IGF2-mRNA-binding protein 2 (IMP2) and negatively regulates IMP2-mediated translation of proliferation factors such as N-Ras and c-Myc [21]. Hypoxia and HIF-1α-induced lincRNA-p21 functions in disrupting the VHL-HIF-1α interaction by binding to HIF-1α, which compromises VHL-mediated HIF-1α ubiquitination and degradation to stabilize HIF-1α under hypoxic conditions [6].

Despite the well-explored but rarely occurred genetic alterations like mutations can led to the dysregulation of EGFR, the aberrant expression of EGFR in various types of cancers suggests the existence of one or several more general pathophysiological mechanisms for regulating EGFR expression. Whether *MALAT1* regulates EGFR is an interesting pathophysiological question. *MALAT1* and EGFR were both downregulated in response to the depletion of tumor necrosis factor-α-inducible protein 8 (TNFAIP8), but no mechanism was proposed [22]. *MALAT1* is highly expressed in hepatocellular carcinoma (HCC) and acts as competing endogenous RNA (ceRNA) for miR-195. As EGFR is the direct downstream target of miR-195, knockdown of *MALAT1* led to increased free miR-195 and downregulation of EGFR. The identification of *MALAT1*-miR-195-EGFR axis suggested a new mechanism about how *MALAT1* indirectly regulates EGFR in HCC [23]. Another report proposed that overexpression of EGFR by increasing *EGFR* mRNA translation can be induced by the hypoxic microenvironment and activation of hypoxia-inducible factor-2α (HIF-2α) in tumors [24]. Interestingly, a positive *MALAT1*/HIF-2α feedback circuit contributing to carcinogenesis has been identified [25], which suggests *MALAT1* may regulation EGFR expression through HIF-2α-mediated pathway. It is worth to emphasize that hypoxic microenvironment or HIF-2α does not regulate EGFR mRNA expression but its translation [24]. Thus, the indirectly regulation of EGFR expression by *MALAT1* may be performed through miRNAs at the posttranscriptional level or activation of hypoxic regulators at the translational level. In this study, as nucleus-localized *MALAT1* does not regulate EGFR mRNA and the protein synthesis machine localizes in cytoplasm, we assumed that a mediator manipulating the protein synthesis of EGFR is transcriptionally regulated by *MALAT1*. Transcriptomic sequencing and subsequent studies confirmed the role of KTN1 in mediating the regulation of EGFR protein expression by *MALAT1*. Thus, the different indirect regulatory mechanisms proposed in our and other studies may suggest the exact mechanism about how *MALAT1* regulates EGFR is cancer-specific.

As an integral endoplasmic reticulum (ER) membrane protein, KTN1 belongs to the kinectin protein family and serves as a receptor for molecular motor kinesin to facilitate in microtubule-based vesicle transportation and membrane trafficking [26]. This protein also binds translation elongation factor-delta and may be involved in the assembly of the elongation factor-1 complex [16]. In this study, the drastic downregulation of EGFR protein expression after KTN1 knockdown suggested that KTN1 acts as the critical mediator in the *MALAT1*-KTN1-EGFR axis for promoting cSCC progression. Our finding is in line with the function of ER-localized KTN1 in regulating protein synthesis [16] and suggested an open question: how does KTN1 interact with the protein translation machinery to influence ER-to-Golgi-regulated protein processing flux and determine the final protein output like EGFR? Especially, this regulatory axis is also existed in cervical cancer and melanoma cells, which indicates the conservation of this mechanism at least in several types of cancers checked in this study.

First discovered in the 1980s, c-MYC is a pivotal regulator in tumorigenesis of up to 70% of all cancers [27]. Elevated expression of c-MYC predicts aggressive disease and a poor clinical outcome in many cancers [28] as well as in cSCC [29]. c-MYC protein can dimerize with MAX to the E-box element [30] and acts as a global regulator involved in cell cycle regulation, mitochondrial function, metabolism, protein synthesis, and ribosome biogenesis [31, 32]. Several reports have shown that c-MYC can bind to the promoter regions of LncRNAs including the imprinted maternally expressed transcript *H19* and colon cancer-associated transcripts 1 (*CCAT1*), and transcriptionally regulate their expression [33, 34]. In addition, c-MYC also physically associates with a LncRNA, prostate cancer gene expression marker 1 (*PCGEM1*), to transcriptionally promote metabolism by enhancing multiple metabolic pathways [35]. In this study, c-MYC is identified as key interacting partner of *MALAT1* to bind the promoter region of *KTN1* and regulate its expression. Interestingly, c-MYC was also reported to transcriptionally activated *MALAT1* by binding to *MALAT1* promoter, which contributes to a novel *SYK/c-MYC/MALAT1* pathway to promote Ewing Sarcoma [36]. Such findings together with our findings suggest that c-MYC not only epigenetically transactivates *MALAT1* expression, but also directly interacts with *MALAT1* to promote KTN1 expression for enhancing EGFR protein expression.

Collectively, our findings revealed a novel mechanism wherein *MALAT1* interacts with c-MYC to transactivate *KTN1* for enhancing EGFR protein expression, which finally contributes to the development of cSCC (Fig. 6g). The positive regulatory axis of *MALAT1-*KTN1-EGFR identified in this study may provide novel drug targets for anti-cSCC therapy.

## Materials and methods

Details for tissue samples, Ultraviolet B treatment, cell proliferation assay, colony formation assay, wound-healing assay, apoptosis analysis, cell migration assay, cell invasiveness assay, DNA constructs, in vitro transcription, in situ hybridization (ISH), RNA-sequencing, RNA immunoprecipitation (RIP) assay, RNA pull-down assay, ChIP-qPCR analysis, electrophoretic mobility shift assay (EMSA), chromatin isolation by RNA purification (ChIRP) assay, xenograft mouse model, immunoblotting, and immunohistochemistry assays are included in Supporting Information.

### Cell culture

cSCC lines A431, HSC-1, and HSC-5 (HonSun Biological Co. Ltd.), the human benign epidermal keratinocyte cell line HaCaT (China Center for Type Culture Collection), cervical cancer cell line Hela and melanoma cell line A375 (CellCook Biotech. Co. Ltd.) were cultured in Dulbecco’s modified Eagle medium (DMEM) supplemented with 10% fetal bovine serum (Invitrogen) and maintained at 37 °C with 5% CO_2_ in a humidified atmosphere. All the cell lines used in this study were authenticated using short tandem repeat (STR) typing and tested for excluding mycoplasma contamination. The results of authentication of cell lines used in this study by STR typing were placed in Supplementary Materials.

### RNA isolation and real-time quantitative PCR

Total RNAs from cells were extracted using TRIzol reagent (Life Technologies) according to the manufacturer’s instructions. cDNAs were prepared using MMLV Reverse Transcriptase (Life Technologies) and Oligo(dT)_20_ primer. mRNA expression analysis was performed using SYBR Green Master Mix (Life Technologies) on a LightCycler 96 Detection System (Roche) using *GAPDH* for normalization. Primers used in this study are listed in Supplementary Table 4.

### Statistical analysis

The sample size chosen to ensure adequate power to detect a pre-specified effect size was determined as previously described [37, 38]. All results are presented as the mean ± s.d. or mean ± s.e. as indicated in figure captions. The data in each group were analyzed using the unpaired, two-tailed Student’s *t*-test or one-way ANOVA test. All data were analyzed for normal distribution and homogeneity of variance tests and considered to reflect significant differences if *P < 0.05, **P < 0.01, ***P < 0.001.

## Supplementary information

SUPPLEMENTAL MATERIAL

Supplementary Table 1

Supplementary Table 2

Supplementary Table 3

Supplementary Table 4

## References

[1] Lomas A, Leonardi‐Bee J, Bath‐Hextall F (2012). A systematic review of worldwide incidence of nonmelanoma skin cancer. Br J Dermatol.

[2] Cheng J, Yan S (2016). Prognostic variables in high‐risk cutaneous squamous cell carcinoma: a review. J Cutan Pathol.

[3] Lee CS, Bhaduri A, Mah A, Johnson WL, Ungewickell A, Aros CJ (2014). Recurrent point mutations in the kinetochore gene KNSTRN in cutaneous squamous cell carcinoma. Nat Genet.

[4] Gutschner T, Diederichs S (2012). The hallmarks of cancer: a long non-coding RNA point of view. RNA Biol.

[5] Gupta RA, Shah N, Wang KC, Kim J, Horlings HM, Wong DJ (2010). Long non-coding RNA HOTAIR reprograms chromatin state to promote cancer metastasis. Nature.

[6] Yang F, Zhang H, Mei Y, Wu M (2014). Reciprocal regulation of HIF-1α and lincRNA-p21 modulates the Warburg effect. Mol Cell.

[7] Ji P, Diederichs S, Wang W, Böing S, Metzger R, Schneider PM (2003). MALAT-1, a novel noncoding RNA, and thymosin β4 predict metastasis and survival in early-stage non-small cell lung cancer. Oncogene.

[8] Xu C, Yang M, Tian J, Wang X, Li Z (2011). MALAT-1: a long non-coding RNA and its important 3’end functional motif in colorectal cancer metastasis. Int J Oncol.

[9] Gutschner T, Hämmerle M, Diederichs S (2013). MALAT1—a paradigm for long noncoding RNA function in cancer. J Mol Med.

[10] Gutschner T, Hämmerle M, Eißmann M, Hsu J, Kim Y, Hung G (2013). The noncoding RNA MALAT1 is a critical regulator of the metastasis phenotype of lung cancer cells. Cancer Res.

[11] Hu Q, Kwon YS, Nunez E, Cardamone MD, Hutt KR, Ohgi KA (2008). Enhancing nuclear receptor-induced transcription requires nuclear motor and LSD1-dependent gene networking in interchromatin granules. Proc Natl Acad Sci.

[12] Chen G, Wang Z, Wang D, Qiu C, Liu M, Chen X (2012). LncRNADisease: a database for long-non-coding RNA-associated diseases. Nucl Acids Res.

[13] Wang J, Zhao L, Lin P, Su X, Chen S, Huang L (2014). GenCLiP 2.0: a web server for functional clustering of genes and construction of molecular networks based on free terms. Bioinformatics.

[14] Merlino G, Xu YH, Ishii S, Clark AJL, Semba K, Toyoshima K (1984). Amplification and enhanced expression of the epidermal growth factor receptor gene in A431 human carcinoma cells. Science.

[15] Arun G, Diermeier S, Akerman M, Chang KC, Wilkinson JE, Hearn S (2016). Differentiation of mammary tumors and reduction in metastasis upon Malat1 lncRNA loss. Genes Dev.

[16] Ong L, Lin P, Zhang X, Chia S, Yu H (2006). Kinectin-dependent assembly of translation elongation factor-1 complex on endoplasmic reticulum regulates protein synthesis. J Biol Chem.

[17] Loots GG, Ovcharenko I (2004). rVISTA 2.0: evolutionary analysis of transcription factor binding sites. Nucl Acids Res.

[18] Schmitz SU, Grote P, Herrmann BG (2016). Mechanisms of long noncoding RNA function in development and disease. Cell Mol Life Sci.

[19] El-Abaseri TB, Fuhrman J, Trempus C, Shendrik I, Tennant RW, Hansen LA (2005). Chemoprevention of UV light-induced skin tumorigenesis by inhibition of the epidermal growth factor receptor. Cancer Res.

[20] Lewis CM, Glisson BS, Feng L, Wan F, Tang X, Wistuba II (2012). A phase II study of gefitinib for aggressive cutaneous squamous cell carcinoma of the head and neck. Clin Cancer Res.

[21] Gong C, Li Z, Ramanujan K, Clay I, Zhang Y, Lemire-Brachat S (2015). A long non-coding RNA, LncMyoD, regulates skeletal muscle differentiation by blocking IMP2-mediated mRNA translation. Dev Cell.

[22] Day TF, Mewani RR, Starr J, Li X, Chakravarty D, Ressom H (2017). Transcriptome and proteome analyses of TNFAIP8 knockdown cancer cells reveal new insights into molecular determinants of cell survival and tumor progression. Methods Mol Biol.

[23] Liu D, Zhu Y, Pang J, Xie W, Feng X, Guo Y (2018). Knockdown of long non‐coding RNA MALAT1 inhibits growth and motility of human hepatoma cells via modulation of miR‐195. J Cell Biochem.

[24] Franovic A, Gunaratnam L, Smith K, Robert I, Patten DA, Lee S (2007). Translational up-regulation of the EGFR by tumor hypoxia provides a nonmutational explanation for its overexpression in human cancer. Proc Natl Acad Sci USA.

[25] Luo F, Sun B, Li H, Xu Y, Liu Y, Liu X (2016). A MALAT1/HIF-2α feedback loop contributes to arsenite carcinogenesis. Oncotarget.

[26] Kumar J, Yu H, Sheetz MP (1995). Kinectin, an essential anchor for kinesin-driven vesicle motility. Science.

[27] Taub R, Kirsch I, Morton C, Lenoir G, Swan D, Tronick SR (1982). Translocation of the c-MYC- gene into the immunoglobulin heavy-chain locus in human Burkitt’s lymphoma and murine plasmacytoma cells. Proc Natl Acad Sci USA.

[28] Eilers M, Eisenman RN (2008). Myc’s broad reach. Genes Dev.

[29] Lohcharoenkal W, Harada M, Loven J, Meisgen F, Landen NX, Zhang L (2016). MicroRNA-203 inversely correlates with differentiation grade, targets c-MYC and functions as a tumor suppressor in cSCC. J Invest Dermatol.

[30] Blackwood EM, Eisenman RN (1991). Max-A-helix-loophelix zipper protein that forms a sequence-specific DNA binding complex with myc. Science.

[31] Zeller KI, Zhao X, Lee CWH, Chiu KP, Yao F, Yustein JT (2006). Global mapping of c-Myc binding sites and target gene networks in human B cells. Proc Natl Acad Sci USA.

[32] Dang CV, Donnell KAO, Zeller KI, Nguyen T, Osthus RC, Li F (2006). The c-Myc target gene network. Semin Cancer Biol.

[33] Barsytelovejoy D, Lau SK, Boutros PC, Khosravi F, Jurisica I, Andrulis IL (2006). The c-Myc oncogene directly induces the H19 noncoding RNA by allele-specific binding to potentiate tumorigenesis. Cancer Res.

[34] Yang F, Xue X, Bi J, Zheng L, Zhi K, Gu Y (2013). Long noncoding RNA CCAT1, which could be activated by c-Myc, promotes the progression of gastric carcinoma. J Cancer Res Clin Oncol.

[35] Hung C, Wang L, Yu Y, Chen H, Srivastava S, Petrovics G (2014). A long noncoding RNA connects c-Myc to tumor metabolism. Proc Natl Acad Sci USA.

[36] Sun H, Lin DC, Cao Q, Pang B, Gae DD, Lee VK (2017). Identification of a novel SYK/c-MYC/MALAT1 signaling pathway and its potential therapeutic value in ewing sarcoma. Clin Cancer Res.

[37] Button KS, Ioannidis JPA, Mokrysz C, Nosek BA, Flint J, Robinson ESJ (2013). Power failure: why small sample size undermines the reliability of neuroscience. Nat Rev Neurosci.

[38] Krzywinski M, Altman N (2013). Points of significance: Power and sample size. Nat Methods.

