# Supplementary material for: MALAT1-KTN1-EGFR regulatory axis promotes the development of cutaneous squamous cell carcinoma
Source: Cell Death Differ. 2019 Jan 25;26(10):2061–73. doi: 10.1038/s41418-019-0288-7 (PMC6748142; doi:10.1038/s41418-019-0288-7)
Supplement: Supplementary file 1 — SUPPLEMENTAL MATERIAL [file 41418_2019_288_MOESM1_ESM.pdf]

Supplemental Materials and Methods

Ultraviolet B treatment. For UVB exposure, HaCaT cells were washed twice with PBS and exposed to UV (irradiating power:1.65 μW·cm^{-2}; wave length: 310 nm; UV bulbs: Shanghai Gucun Co. Ltd.) at a dose of 30 mJ·cm^{-2}. The treated cells were collected into RNA isolation after 30 min, and 1, 2, 3, 4, 5, 6, 12, and 16 h, and used for expression quantification of *MALAT1* by qRT-PCR.

Cell proliferation and colony forming assays. A431 cells (4000 per well) cultivated on 96-well plates were transfected with ASOs or siRNAs, and cell proliferation were detected after 0, 24, 48, and 72 h using a cell counting kit (TransGen Biotech) at 450 nm as described in the manual. For the colony forming assay, transfected cells were incubated in 6-well plates at 1000 cells per well, which were maintained in DMEM. Medium was replaced 2 times. At day 7, cells were collected after being washed twice with PBS and fixed in 4% paraformaldehyde for 30 min. Finally, the cells were stained with 0.1% crystal violet. Visible colonies were photographed and counted.

Apoptosis assay. A431 cells were seeded on a 60-mm dish and transfected with *MALAT1* ASO1, ASO2, or NC ASO and cultured for 48 h. TransDetect Annexin V-FITC/PI cell apoptosis detection kit (TransGen Biotech) was applied according to manufacturer protocols. Cell apoptosis was detected and quantified by Guava easyCyte Flow Cytometry System (Merk Millipore).

Wound healing assay. A431 cells were seeded in 6-well cell culture plates and transfected

with *MALAT1* ASO1, ASO2, or NC ASO. A yellow pipette tip was used to make a straight scratch to stimulate a wound. The distances travelled (mobility) were measured at 0, 6, and 12 h after scratching.

Cell migration assay. To assess cell migration, 2.0 × 10^5 A431 cells transfected with NC or *MALAT1* ASO1 or ASO2 were seeded into the 8-μm upper chambers of 12-well plates for transwell assay (Cat. No. MCEP24H48, Merk Millipore) in serum-free DMEM. During culture at 37 °C for 48 h, the cells in the upper chambers were attracted by the culture media in the lower chamber, through chemoattractants provided by the included 10% FBS. The chambers were washed with PBS twice and fixed with 3.7% formaldehyde. Cells were permeabilised using 100% methanol at RT, stained with 0.1 crystal violet, and observed using a microscope after being scraped off with cotton swabs.

Cell invasiveness assay. For assessment of invasive ability, Matrigel-coated chambers (Cat. No. 354480, BD Biosciences) were used to culture transfected A431 cells, of which 1.0 × 10^5 cells were seeded into the upper chambers. Other treatments were performed as described in kit manual.

Tissue samples. cSCC samples were obtained from patients diagnosed with cutaneous SCC from January 2009 to August 2016 in the departments of dermatology, pathology, and oncology at Nanfang Hospital and Zhujiang Hospital, affiliated to Southern Medical University. Briefly, a total of 80 specimens of cSCC (54 samples from men and 26 from women) and 10 specimens from normal skin were included in the analysis. Approximately 74%

of the patients were >50 years of age. Fresh samples obtained during surgery were immediately frozen in liquid nitrogen for subsequent total RNA extraction, protein extraction, and paraffin embedding. Tumors were classified according to the SCC Broders Pathological Classification [1]: grade I (well differentiated) with 75–100% differentiated cells, grade II (moderately differentiated) with 50–75% differentiated cells, and grades III and IV (poorly differentiated) with 0–50% differentiated cells. Of the 113 cases, 61 were well differentiated (54.0%), 36 were moderately differentiated (31.9%), and 16 were poorly differentiated (14.2%).

DNA constructs. The full-length *MALAT1* sequence was analysed using the Vienna RNA secondary structure server [2] and separated into 5 fragments based on the mountain plot of minimum free energy analysis (Supporting Information Fig. 2). Fragment #1 (1–1882 bp), #2 (1883–3366 bp), #3 (3367–4941), #4 (4942–6637) and #5 (6638–8443 bp) *MALAT1* domains were PCR amplified and cloned with the pEASY-Blunt Zero Cloning Kit (TransGen Biotech), whereas enhanced green fluorescent protein (EGFP) was cloned into the XbaI site of pcDNA3.1(+) for *in vitro* transcription as described in a previous study [3].

***In vitro* transcription.** For generating sense transcripts of the deletion mutant fragments of *MALAT1*, *in vitro* transcription was performed using a MAXIscript T7/T3 kit (Ambion) after linearisation of the plasmids.

***In situ* hybridisation (ISH).** Antisense single-stranded DNA probe (Supporting Information Table 4) was synthesized and labelled with digoxigenin (DIG) (Roche). The pre-hybridization,

hybridization, Anti-Digoxigenin-AP Fab fragments (Roche, Cat. No. 11093274910) incubation (1:2000), and colour reactions with NBT/BCIP were performed as described in previous studies [3, 4]. NBT/BCIP produces a purple blue precipitate and supports long exposure times.

RNA-sequencing. Sequencing was performed at Beijing Novogene Co., Ltd. using the Illumina HiSeq 2500 instrument. RNA-Seq data was aligned to the reference genome (human assembly GRCh37/hg19) using Tophat2 (http://ccb.jhu.edu/software/tophat). HTSeq (http://www-huber.embl.de/HTSeq) was then applied on the aligned data set to determine differentially expressed genes with a 'significant' status. The GO and KEGG analysis of the differentially expressed genes was performed using DAVID (http://david.abcc.ncifcrf.gov/). All the generated RNA-seq datasets can be found in the Gene Expression Omnibus (GEO) database, www.ncbi.nlm.nih.gov/geo (Accession codes: GSE100399, GSM2680311 and GSM2680312).

RNA immunoprecipitation (RIP) assay. RIP was performed as described [3, 5]. 5 μg antibodies against c-MYC (CST) or isotype IgG (Merck Millipore) as a negative control was used.

RNA pull-down assay. RNA pull-down was performed as previous described [3, 5]. Biotinylated *MALAT1* fragments and *LacZ* RNAs were synthesized using a MAXIscript T7/T3 *in vitro* transcription kit (Ambion) and Biotin RNA labelling Mix (Roche).

ChIP-qPCR analysis. ChIP experiments were performed as recommended by the EZ ChIP™ Chromatin Immunoprecipitation kit manual (Cat. no. 17-371, Merck Millipore) using 5 μg antibodies against c-MYC (CST) or isotype IgG (Merck Millipore) used as a negative control. Relative enrich of c-MYC at KTN1 locus was quantified by qRT-PCR as previously described [3, 5]. Primers used are listed in Supporting Information Table 4.

Electrophoretic mobility shift assay (EMSA). EMSA experiments were carried out using forward strand 5′Cy5-labelled-dsDNA (Life Technologies) as previously described [3]. 100 nM dsDNA probe containing the E-box motif at KTN1 promoter was tested for binding with purified c-MYC protein (Cat. no. ab169901, Abcam). Supershift assay was performed by incubating c-MYC antibody with the above E-box motif-containing probe and c-MYC protein and detecting the formation of supershift bind. The bands were detected using the ODYSSEY infrared imaging system (LI-COR Biosciences).

Chromatin isolation by RNA purification (ChIRP) assay. Anti-sense DNA tiling probes for the selective retrieval of *MALAT1* were designed using an online probe designer at http://singlemoleculefish.com/ and synthesised by Life Technologies Inc. with BiotinTEG at the 3-prime end. The ChIRP assay was performed according to the kit instructions of Magna ChIRP™ RNA Interactome Kits (Merck Millipore) and also with reference to previous studies [3, 6]. Specifically, retrieved *MALAT1* RNA and DNA regions expected to bind *MALAT1* were quantified by qRT-PCR as previously described [3, 6].

Xenograft mouse model. Briefly, 1×10^7 cells were subcutaneously implanted into the left

and right flanks of female athymic nude mice (4–5 weeks old). At 8 days after implantation, NC ASO or *MALAT1* ASO was injected into the left or right tumor, respectively; and the injection was repeated every other day. Oligos were prepared by pre-incubating 3 nM ASO per mouse with Lipofectamine 2000 (Life Technologies) for 15 min; injections were made using a final volume of 50 μl in serum free DMEM (Life Technologies). The tumor diameters were measured and recorded every day to generate a tumor growth curve. After tumor growth assessment, the tumors were excised and snap-frozen for RNA and protein extraction or paraffin-embedded for IHC staining.

Immunoblotting and immunohistochemistry assays. Total cell extracts were prepared and assayed by western blot as previously described [3, 7]. The following primary antibodies and dilutions were used: anti-c-MYC (Cell Signaling Technology, #13987, 1:2000), anti-KTN1 (Cell Signaling Technology, #13243, 1:2000), anti-EGFR (Santa Cruz Biotechnology, #sc-03, 1:2000), anti-PI3 Kinase p85 (Cell Signaling Technology, #4292, 1:2000), anti-AKT (Cell Signaling Technology, #4691, 1:2000), anti-Phospho-AKT (Cell Signaling Technology, #13038, 1:2000), anti-mTOR (Cell Signaling Technology, #2983, 1:2000), anti-Phospho-mTOR (Cell Signaling Technology, #2971, 1:2000), anti-STAT3 (abcam, ab68153, 1:2000), anti-Phospho-STAT3 (abcam, ab76315, 1:2000) and anti-GAPDH (Santa Cruz Biotechnology, #sc-25778, 1:5000). The following secondary antibodies were also used: anti-mouse IgG-horseradish peroxidase (HRP) (Santa Cruz Biotechnology, #sc-2005, 1:5000), anti-rabbit IgG-HRP (Santa Cruz Biotechnology, #sc-2357, 1:5000), and anti-goat IgG-HRP (Santa Cruz Biotechnology, #sc-2354, 1:5000). Bound antibodies were visualised using the Luminata Forte Western HRP substrate (Millipore).

IHC staining of formalin-fixed paraffin-embedded cSCC sections or xenograft tumor sections was performed with the following antibodies: anti-c-MYC (Cell Signaling Technology, #13987, 1:100), anti-KTN1 (Cell Signaling Technology, #13243, 1:100), and anti-EGFR (Santa Cruz Biotechnology, #sc-03, 1:100). Stained IHC sections were imaged with an ZEISS Axio Vert.A1 microscope and at least 10 representative images were collected for statistical analysis.

References:

1. Cassarino DS, Derienzo DP, Barr RJ. Cutaneous squamous cell carcinoma: a comprehensive clinicopathologic classification--part two. *J CUTAN PATHOL* 2006 2006-04-01; **33:** 261-279.
2. Gruber AR, Bernhart SH, Lorenz R. The ViennaRNA web services. *Methods Mol Biol* 2015 2015-01-20; **1269:** 307-326.
3. Zhou L, Sun K, Zhao Y, Zhang S, Wang X, Li Y, *et al.*. Linc-YY1 promotes myogenic differentiation and muscle regeneration through an interaction with the transcription factor YY1. *NAT COMMUN* 2015 2015-12-11; **6:** 10026.
4. Obernosterer G, Martinez J, Alenius M. Locked nucleic acid-based in situ detection of microRNAs in mouse tissue sections. *NAT PROTOC* 2007 2007-01-20; **2:** 1508-1514.
5. Tsai MC, Manor O, Wan Y, Mosammaparast N, Wang JK, Lan F, *et al.*. Long noncoding RNA as modular scaffold of histone modification complexes. *SCIENCE* 2010 2010-08-06; **329:** 689-693.
6. Chu C, Qu K, Zhong FL, Artandi SE, Chang HY. Genomic maps of long noncoding RNA occupancy reveal principles of RNA-chromatin interactions. *MOL CELL* 2011 2011-11-18; **44:** 667-678.
7. Zhou L, Wang Y, Ou C, Lin Z, Wang J, Liu H, *et al.*. microRNA-365-targeted nuclear factor I/B transcriptionally represses cyclin-dependent kinase 6 and 4 to inhibit the progression of cutaneous squamous cell carcinoma. *Int J Biochem Cell Biol* 2015 2015-08-01; **65:** 182-191.

Supplementary Figure S1

a

Database Num	Human Gene Symbol	Publications
196	MIR7-3HG	2065
95	H19	1487
282	TERC	979
306	XIST	514
19	BDNF-AS	463
191	MIR17HG	457
220	PCA3	424
178	MEG3	311
173	MALAT1	285
122	KCNQ1OT1	283

b

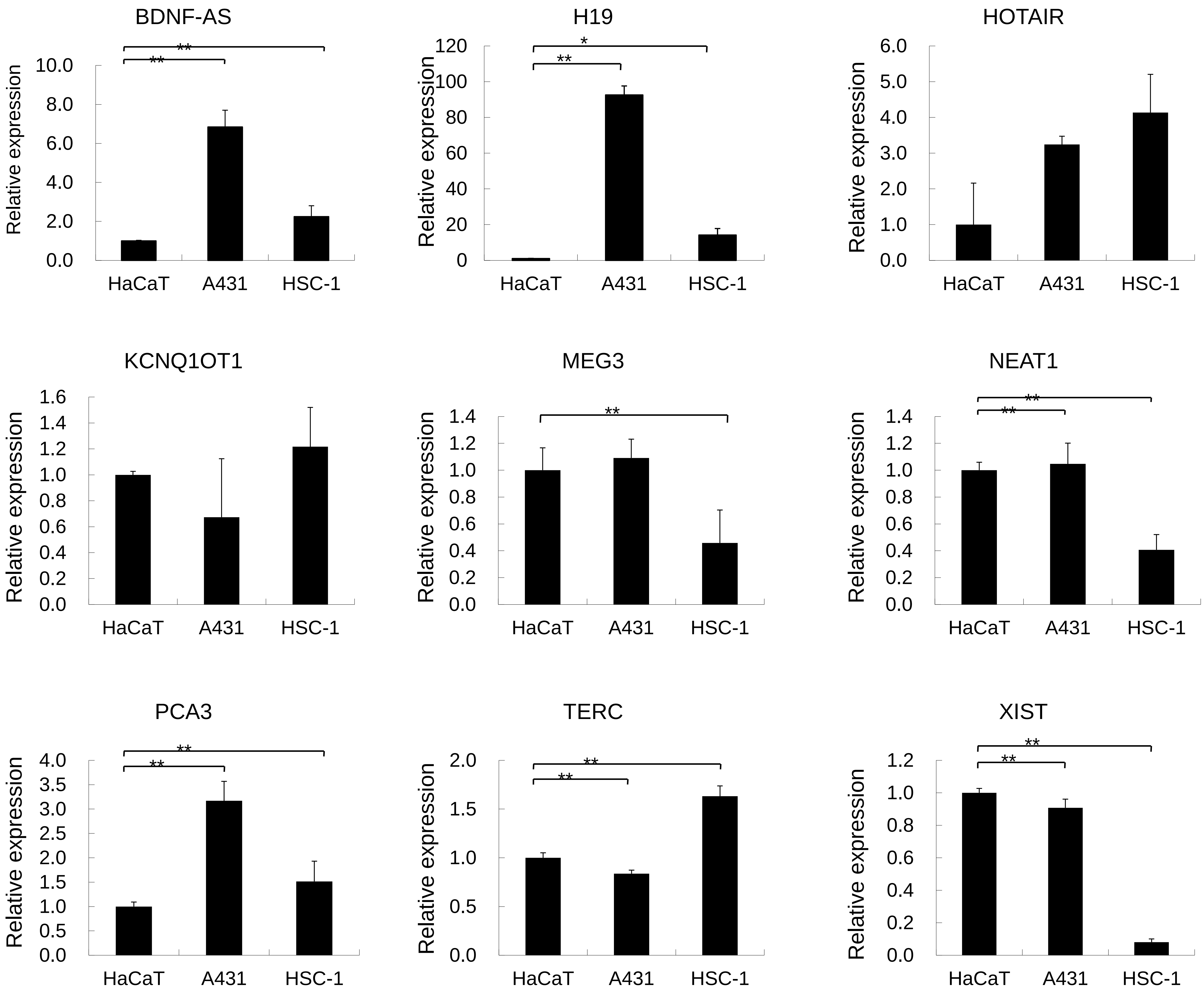

Supplementary Figure S1. Expression of LncRNAs in HaCaT keratinocytes, A431 and HSC-1 cSCC cells. (a) Diseased-related LncRNAs from LncRNADisease database (http://cmbi.bjmu.edu.cn/lncrnadisease) were collected and analysed with GenClip 2.0 (http://ci.smu.edu.cn/GenCLiP2/) to obtained 10 most-studied LncRNAs. (b) Total RNAs were extracted and analysed the above LncRNAs by qRT-PCR. All statistical data represent the average of three independent experiments ±s.d. * $P < 0.05$, ** $P < 0.01$, *** $P < 0.001$.

Supplementary Figure S2

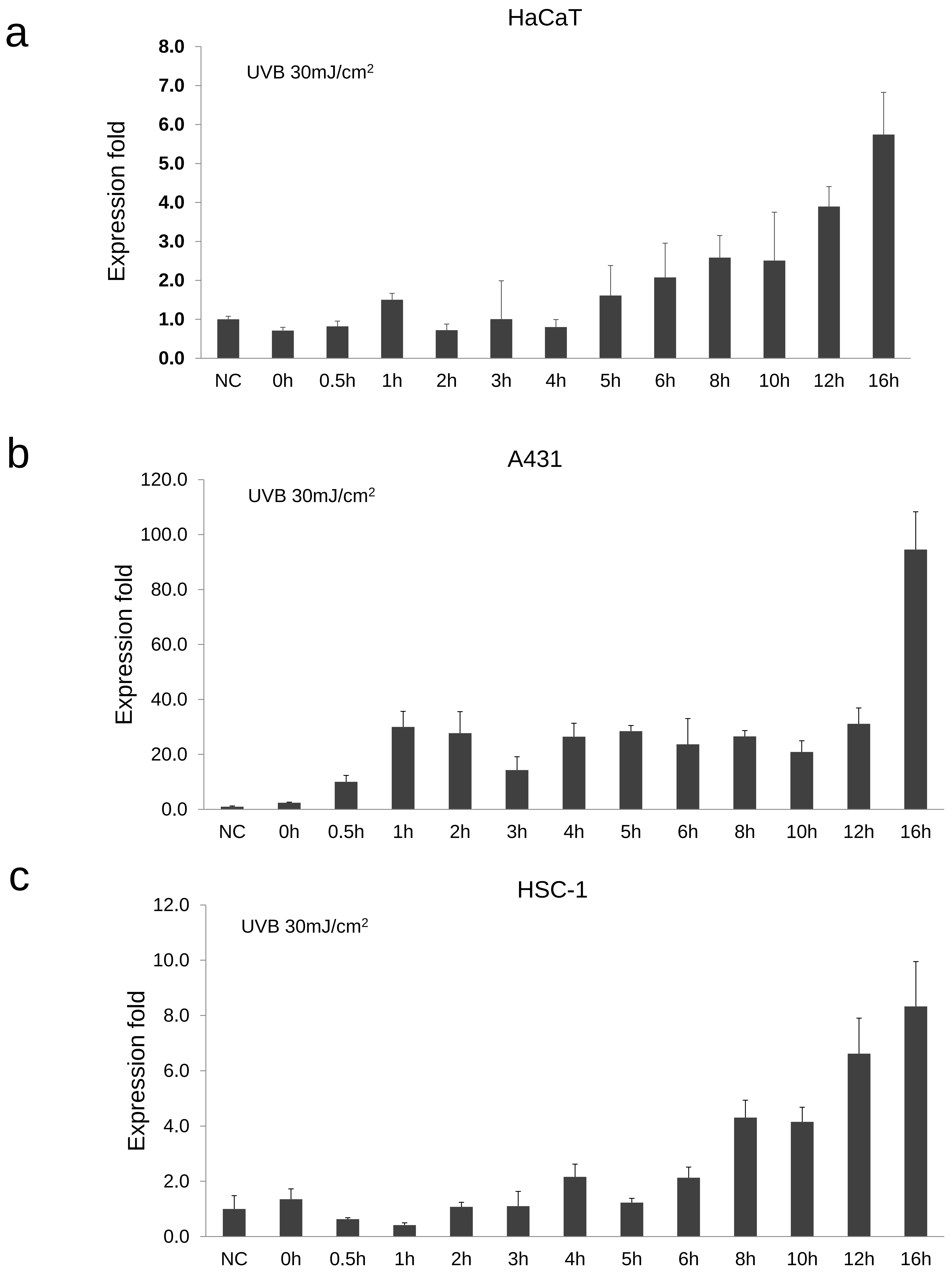

Supplementary Figure S2. *MALAT1* is induced by UV irradiation in keratinocytes and cSCC cell lines. The expression levels of *MALAT1* were gradually upregulated in response to UV radiation in HaCaT keratinocytes (a), and A431 (b) and HSC-1 (c) cSCC cell lines. The above cells were UVB irradiated with dose of 30 mJ·cm^{-2}. Total RNAs were extracted and analyzed by qRT-PCR.

Supplementary Figure S3

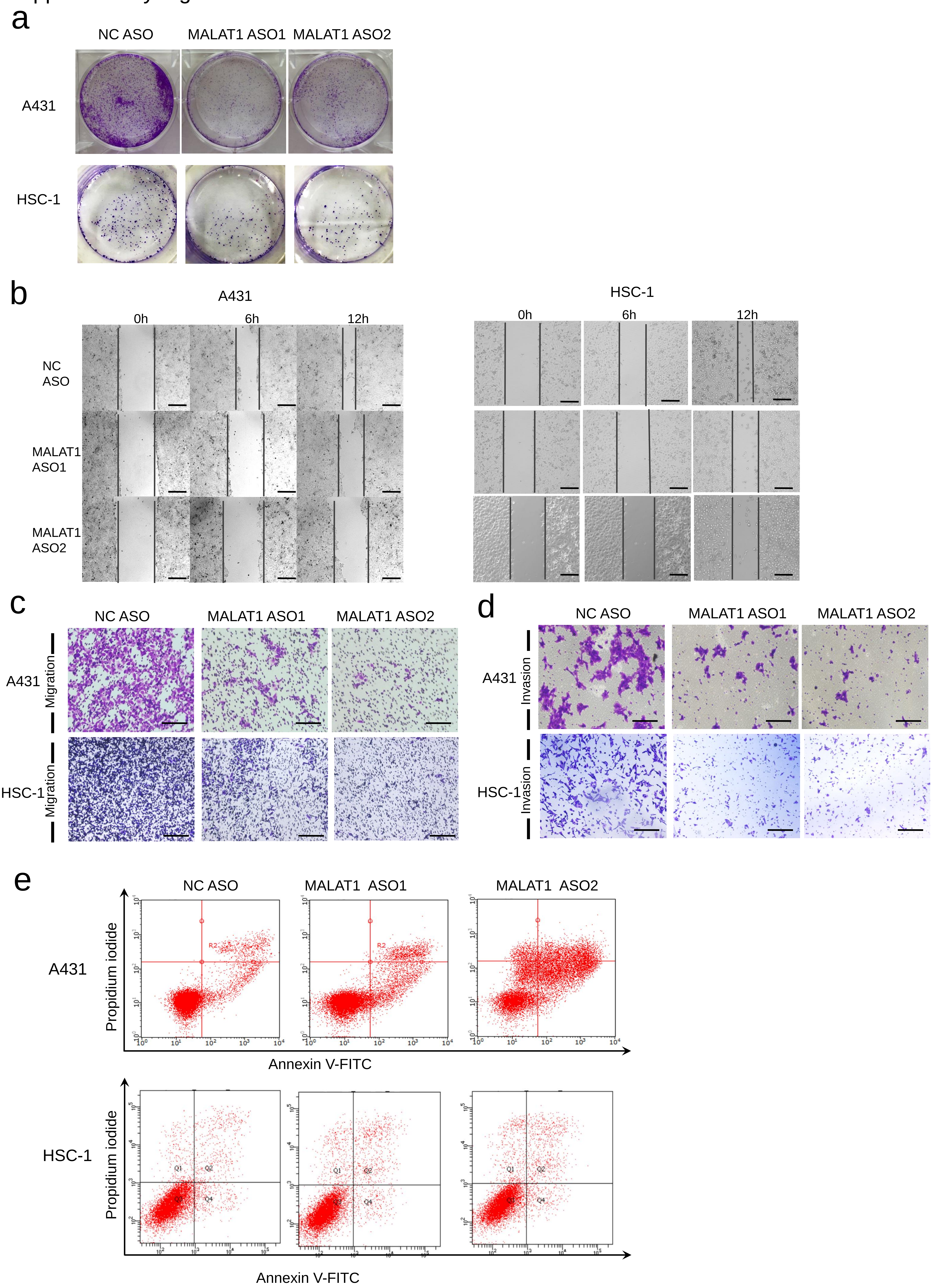

Supplementary Figure S3. Knockdown of *MALAT1* inhibits cell proliferation, mobility, migration, and invasion but promotes apoptosis of A431 and HSC-1 cells. (a) Monolayer colony formation assay showing that *MALAT1* knockdown significantly decreased the colony formation capacity of A431 and HSC-1 cells. (b) Wound healing assay showing that knockdown of *MALAT1* significantly decreased the wound closure ability of A431 and HSC-1 cells at both 24 and 48 h. (c) Transwell assay indicated that *MALAT1* knockdown suppressed A431 and HSC-1 cell motility. (d) Matrigel invasion assay indicated that *MALAT1* knockdown drastically inhibited A431 and HSC-1 cell invasiveness. (e) Flow cytometric analysis using Annexin V/PI staining showing that *MALAT1* silencing significantly increased the apoptosis of A431 and HSC-1 cells. Scale bars, 100 µm.

Supplementary Figure S4

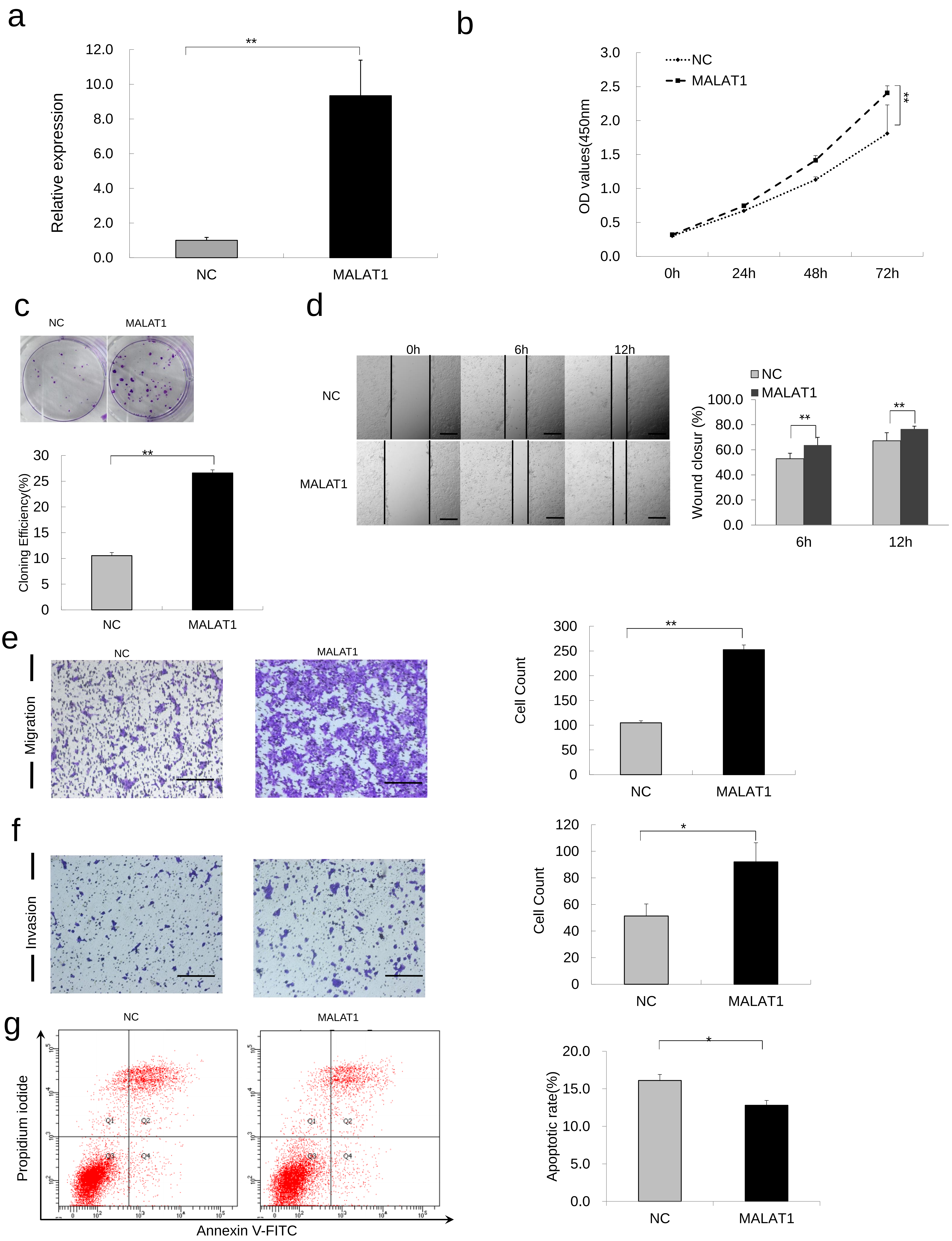

Supplementary Figure S4. Over expression of *MALAT1* promotes cell proliferation, mobility, migration, and invasion but inhibits apoptosis of A431 cells. (a) Over expression of *MALAT1* in A431 cells were determined by qRT-PCR assay. (b) CCK-8 assay determination of A431 cell proliferation in response to *MALAT1* over express. (c) Monolayer colony formation assay showing that *MALAT1* over express significantly increased the colony formation capacity of A431 cells. The colony numbers were counted and recorded. (d) Wound healing assay showing that over express of *MALAT1* significantly increased the wound closure ability of A431 cells at both 6 and 12 h. (e) Transwell assay indicated that over expression of *MALAT1* promoted A431 cell motility. (f) Matrigel invasion assay indicated that over expression of *MALAT1* drastically promoted A431 cell invasiveness. (g) Flow cytometric analysis using Annexin V/PI staining showing that over expression of *MALAT1* significantly decreased the apoptosis of A431 cells (*$P < 0.05$, **$P < 0.01$ with LSD test of One-Way ANOVA). All statistical data represent the average of three independent experiments ±s.d. * $P < 0.05$, ** $P < 0.01$, *** $P < 0.001$. Scale bars, 100 μm.

Supplementary Figure S5

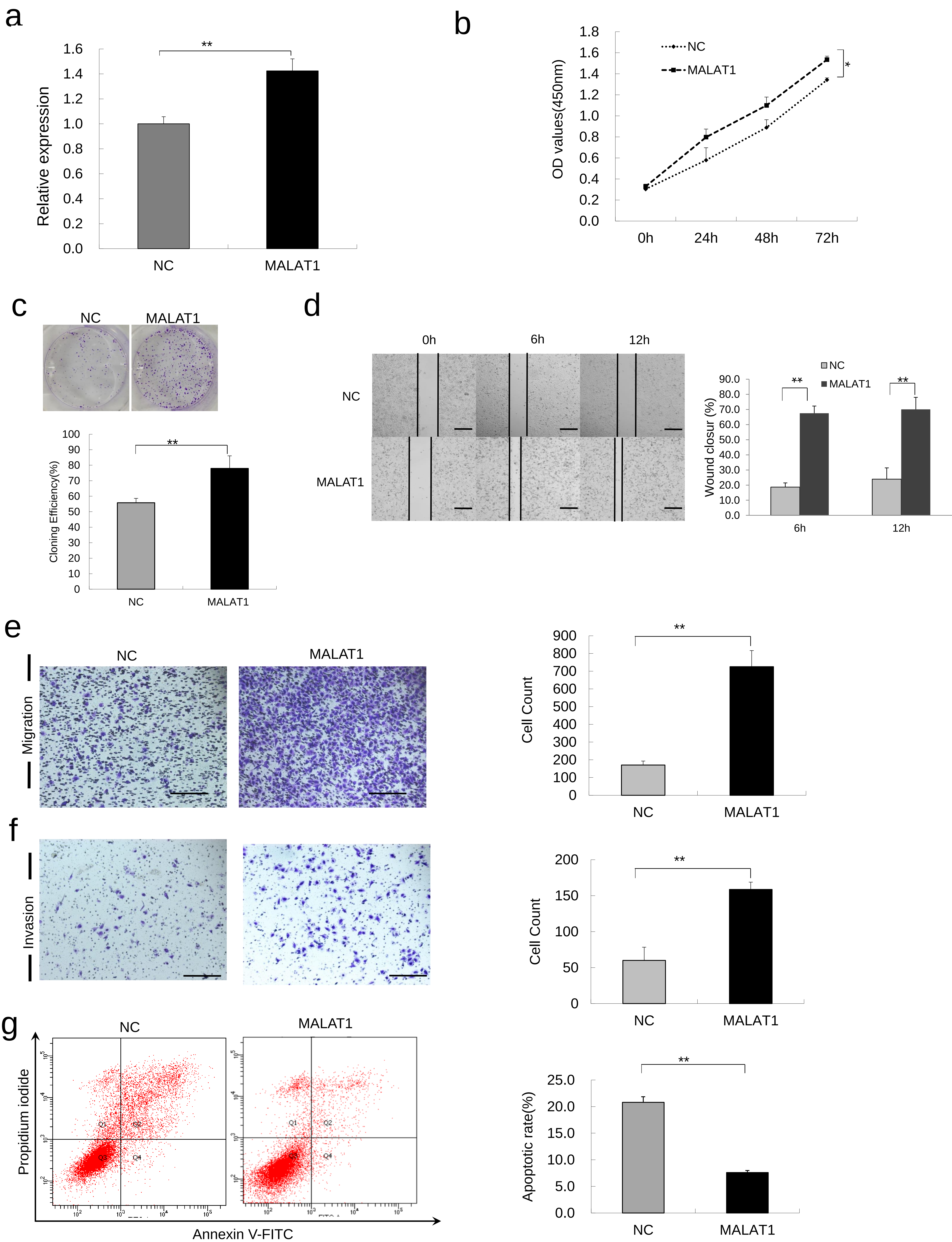

Supplementary Figure S5. Over expression of *MALAT1* promotes cell proliferation, mobility, migration, and invasion but inhibits apoptosis of HSC-1 cells. (a) Over expression of *MALAT1* in HSC-1 cells were determined by qRT-PCR assay. (b) CCK-8 assay determination of HSC-1 cell proliferation in response to *MALAT1* over express. (c) Monolayer colony formation assay showing that *MALAT1* over express significantly increased the colony formation capacity of HSC-1cells. The colony numbers were counted and recorded. (d) Wound healing assay showing that over express of *MALAT1* significantly increased the wound closure ability of HSC-1 cells at both 6 and 12 h. (e) Transwell assay indicated that over expression of *MALAT1* promoted HSC-1 cell motility. (f) Matrigel invasion assay indicated that over expression of *MALAT1* drastically promoted HSC-1 cell invasiveness. (g) Flow cytometric analysis using Annexin V/PI staining showing that over expression of *MALAT1* significantly decreased the apoptosis of HSC-1 cells (*$P < 0.05$, **$P < 0.01$ with LSD test of One-Way ANOVA). All statistical data represent the average of three independent experiments $\pm$s.d. * $P < 0.05$, ** $P < 0.01$, *** $P < 0.001$. Scale bars, 100 μm.

Supplementary Figure S6

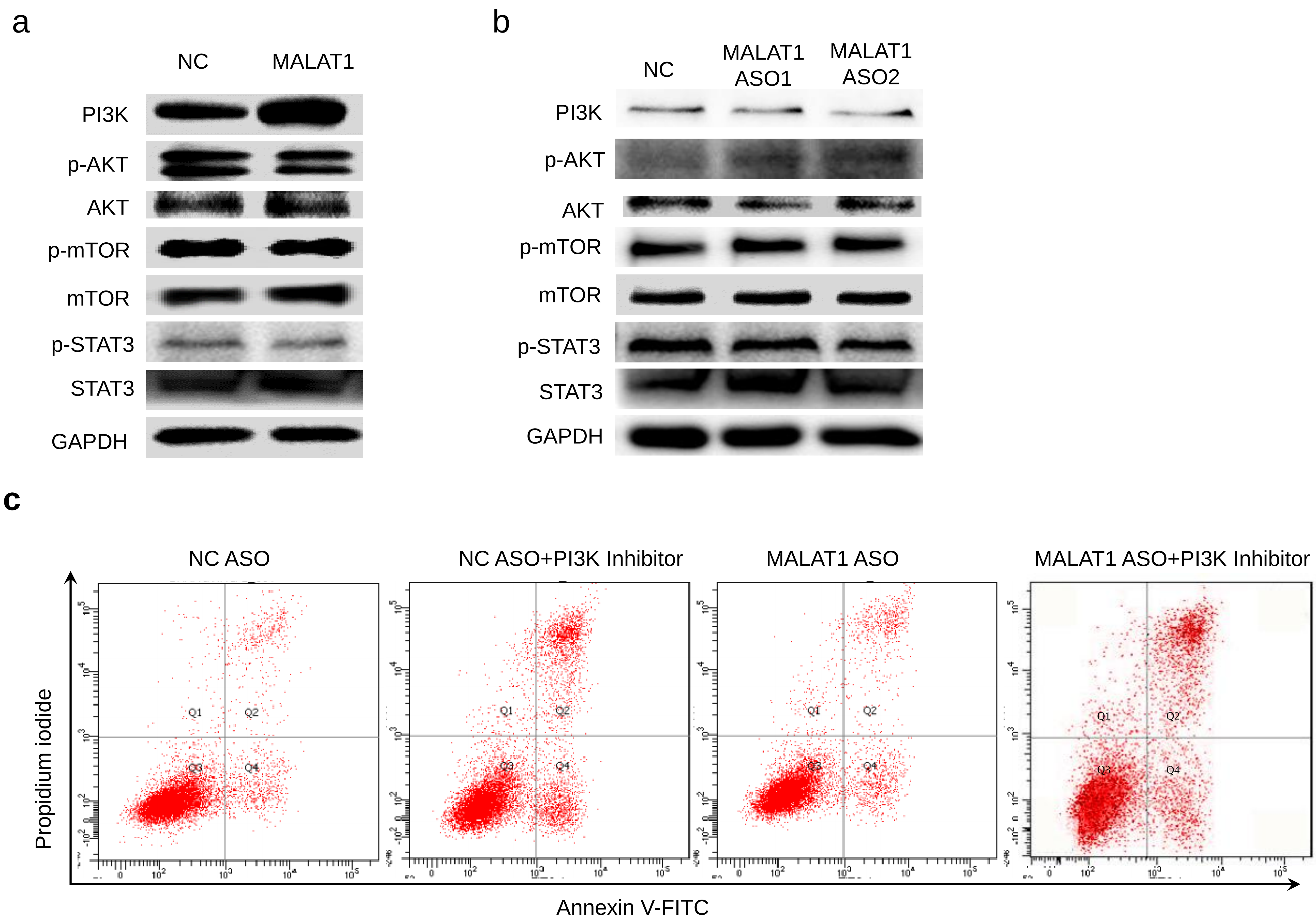

Supplementary Figure S6. *MALAT1* inhibits the apoptosis of A431 cells through PI3K pathway. (a, b) The expression levels of master factors in STAT3, PI3K, AKT and mTOR signaling pathways were examined in A431 cells after *MALAT1* overexpression or knockdown. (c) Flow cytometric analysis using Annexin V/PI staining showing that PI3K inhibitor LY294002 treatment led to much high apoptotic rate in addition to *MALAT1* depletion-induced apoptosis.

Supplementary Figure S7

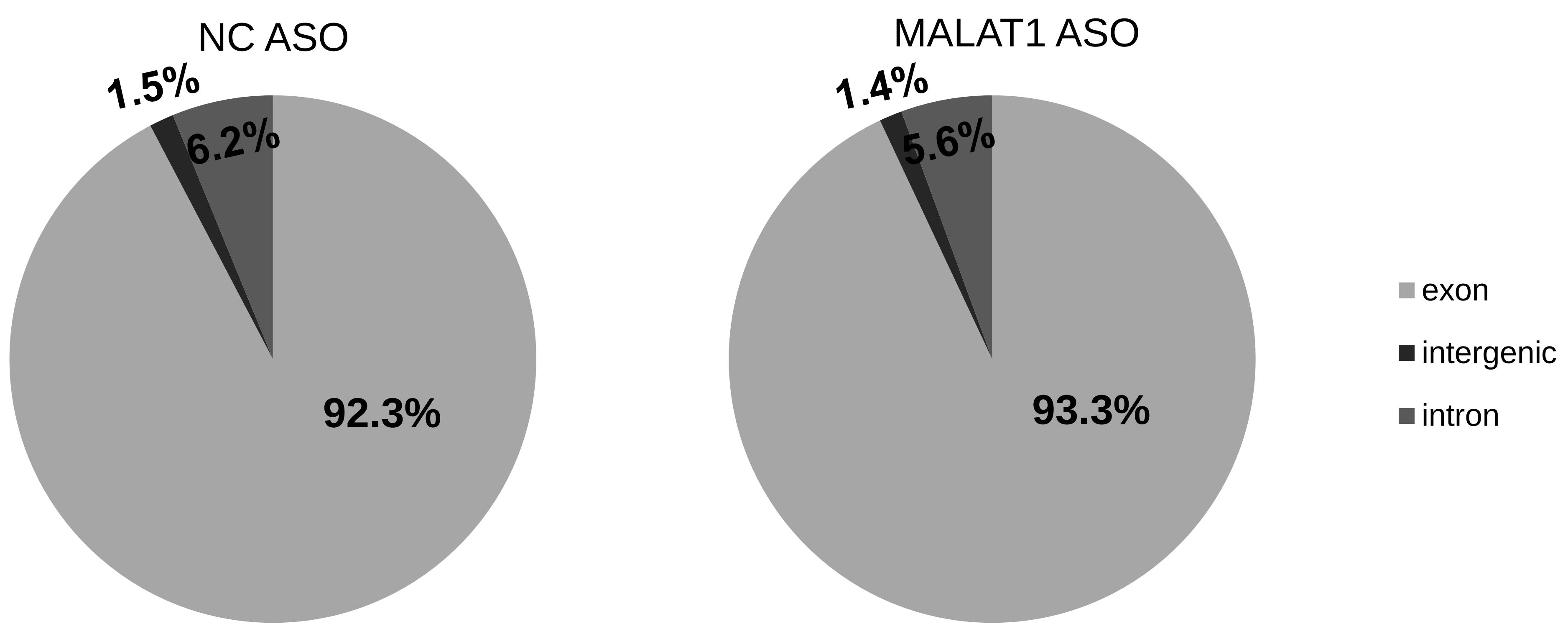

Supplementary Figure S7. Total RNAs were isolated from A431 cells transfected with NC or *MALAT1* ASOs, and subjected to high throughput transcriptomic sequencing. The normalized fragment density was calculated by counting the fragments per kilobase of genomic regions of interests (exon, intron, intergenic regions) per million mapped reads.

Supplementary Figure S8

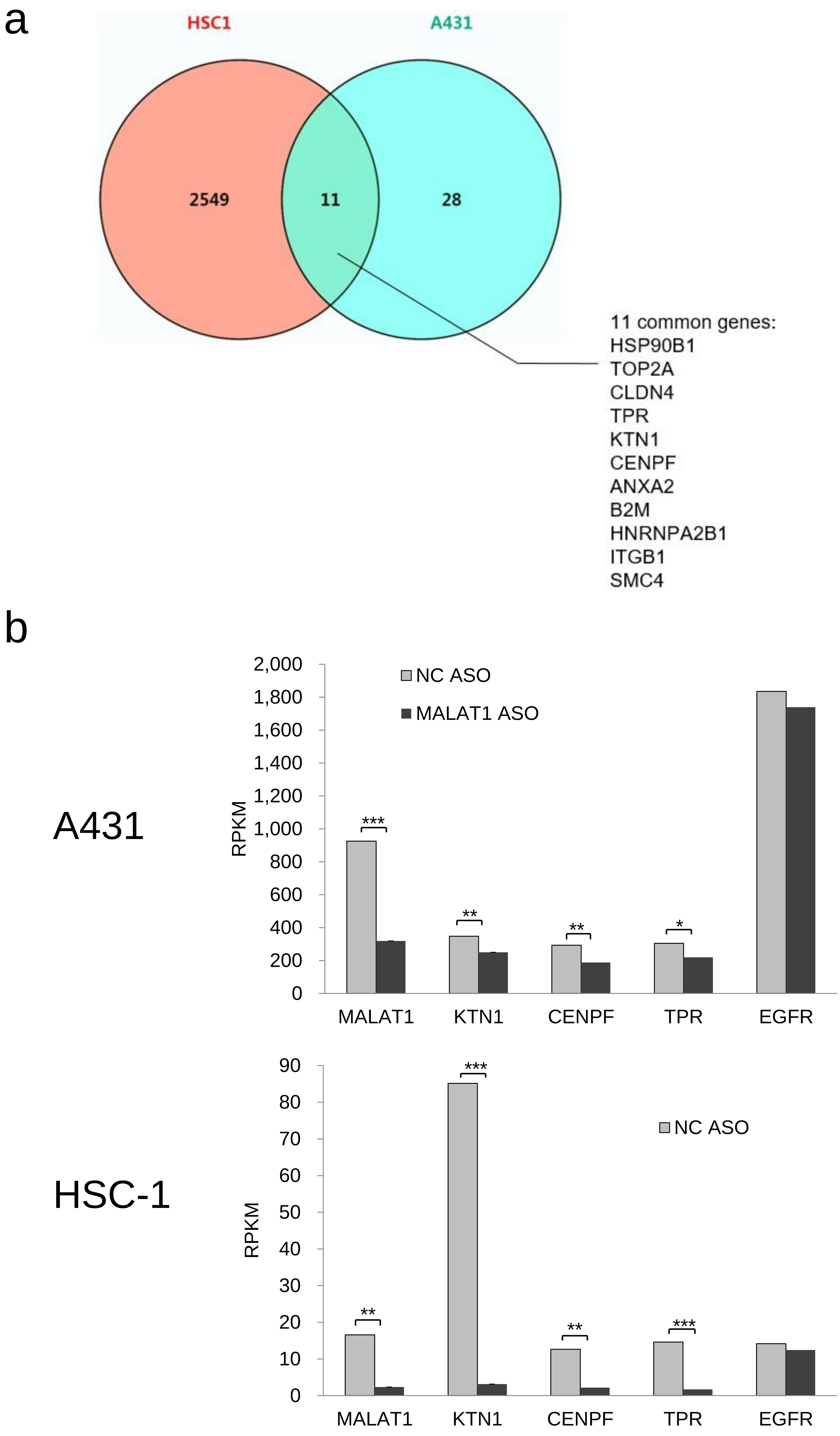

Supplementary Figure S8. Analysis of common genes significantly varied after *MALAT1* depletion. (a) Venn diagram analysis shows the common genes significantly varied after *MALAT1* depletion in both A431 and HSC-1 cell lines. (b) The comparison of the expression levels (RPKM) of *MALAT1*, *KTN1*, *CENPF*, *TPR* and *EGFR* detected by RNA-seq between NC ASO and *MALAT1* ASO treatment.in A431 and HSC-1 cell lines. (*P<0.05, **P<0.01, ***P<0.001)

Supplementary Figure S9

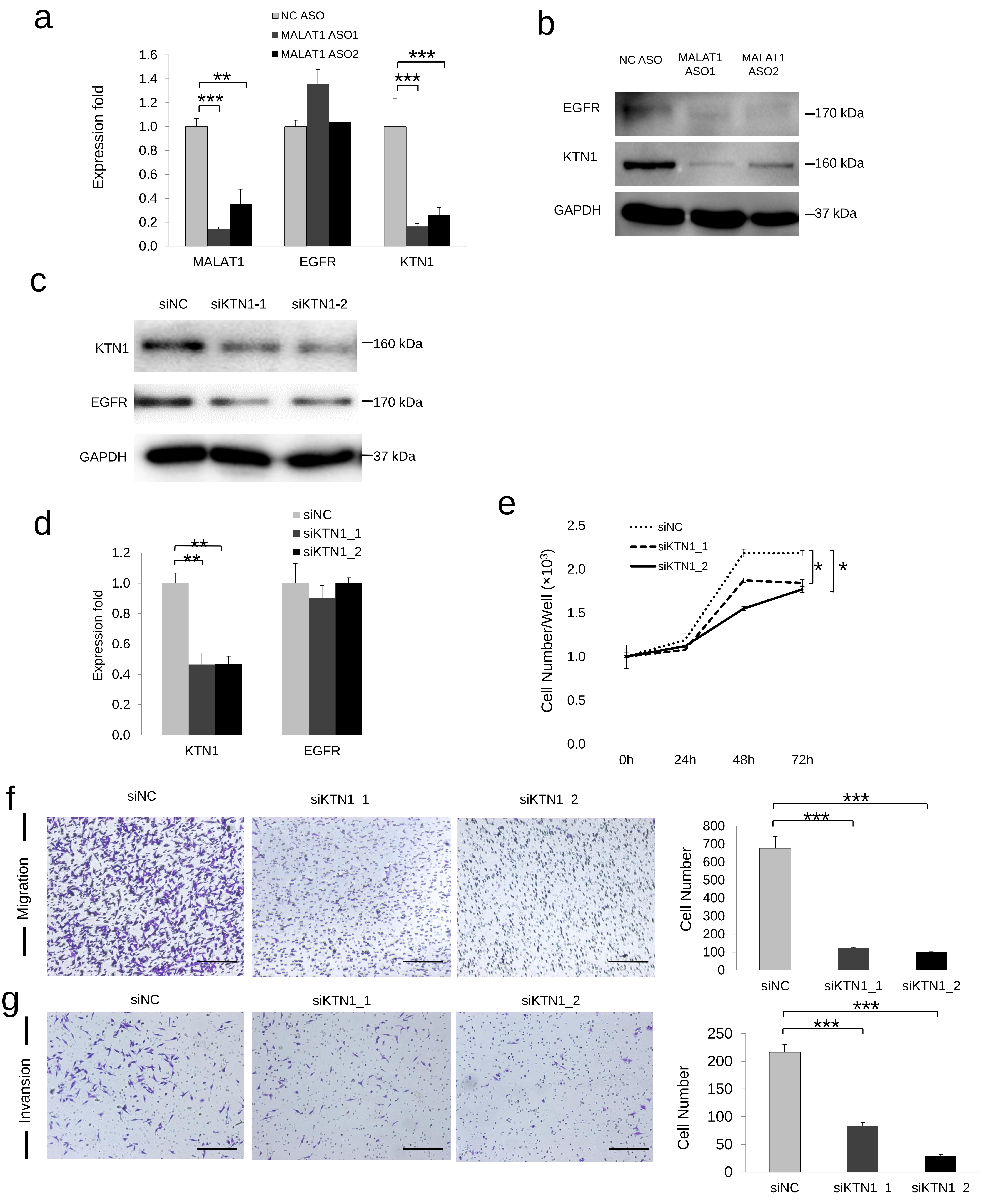

Supplementary Figure S9. KTN1 mediates *MALAT1* regulation of EGFR in another cSCC cell line, HSC-1. (a) RNA expression of *MALAT1* and *KTN1* was examined in HSC-1 cells with two different *MALAT1* ASOs by qRT-PCR. (b) KTN1 protein expression was examined in *MALAT1* depleted HSC-1 cells by western blot, which showed significant loss of KTN1 in response to *MALAT1* knockdown. (c) RNA interference (RNAi) with siRNAs targeting *KTN1* was applied to HSC-1 cells and the protein expression of KTN1 and EGFR was detected using western blot. (d) RNA expression of *KTN1* and *EGFR* was analysed by qRT-PCR to determine the efficiency and specificity of *KTN1* RNA knockdown. (e) CCK-8 assay determination of HSC-1 cell proliferation in response to KTN1 knockdown (with Dunnett T3 test of One-Way ANOVA). (f) Transwell assay of HSC-1 cell motility following KTN1 knockdown. (g) Matrigel invasion assay of HSC-1 cell invasiveness following KTN1 knockdown. All statistical data represent the average of three independent experiments ± s.d. * $P < 0.05$, ** $P < 0.01$, *** $P < 0.001$. Scale bars, 100 μm.

Supplementary Figure S10

a

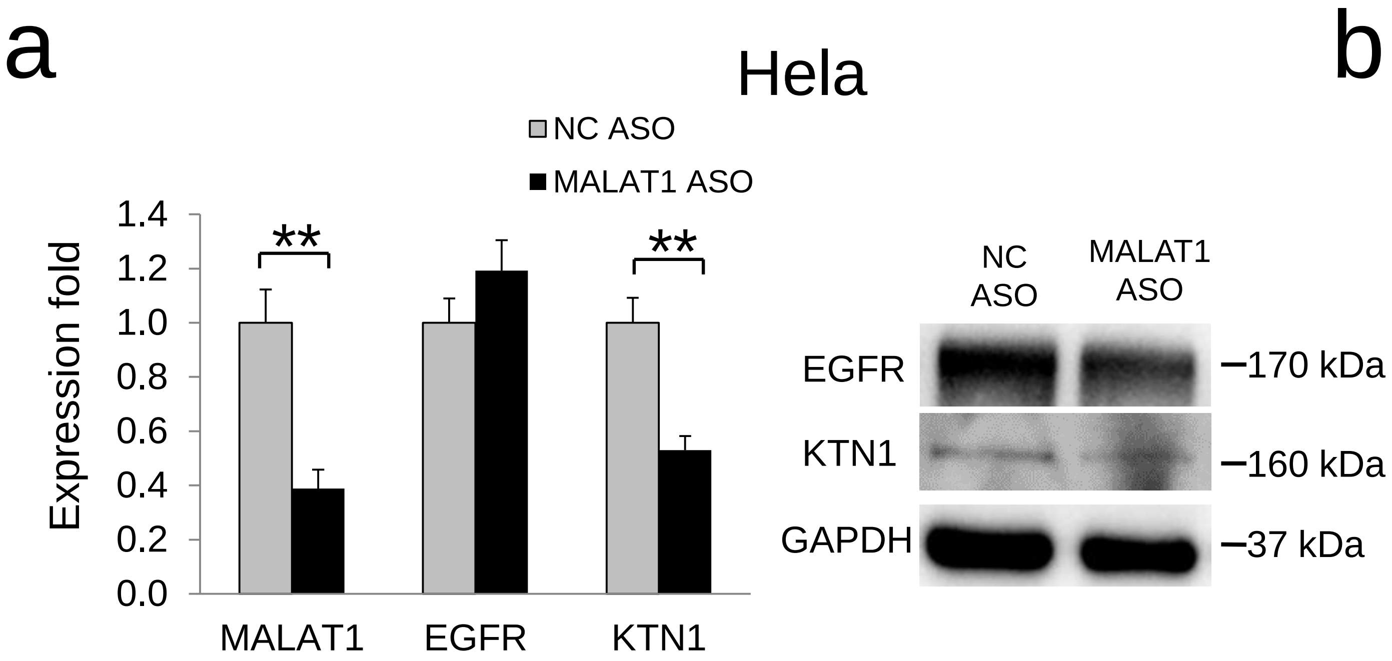

b

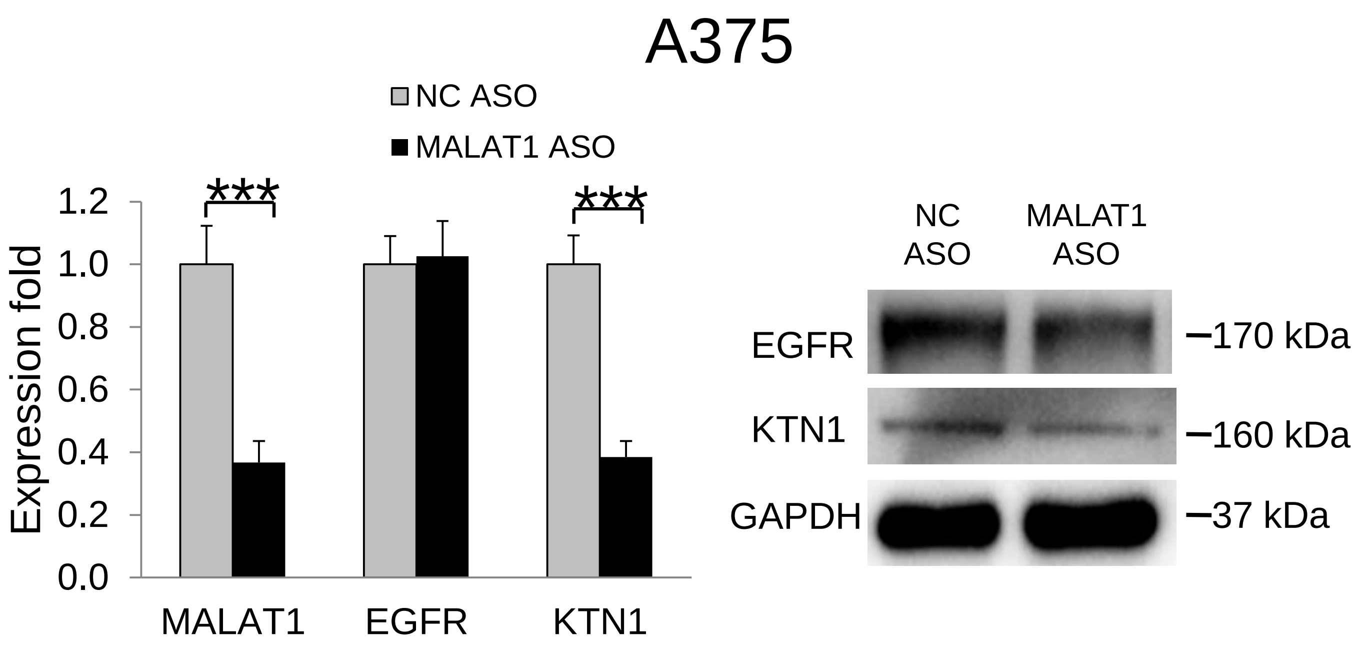

Supplementary Figure S10. *MALAT1* expression was knocked down using *MALAT1* ASOs in Hela (a) and A375 (b) cell lines. Western blot and qRT-PCR was performed to detect gene expression of *MALAT1* and both mRNA and protein expression of KTN1 and EGFR. All statistical data represent the average of three independent experiments ± s.d. * $P < 0.05$, ** $P < 0.01$, *** $P < 0.001$.

Supplementary Figure S11

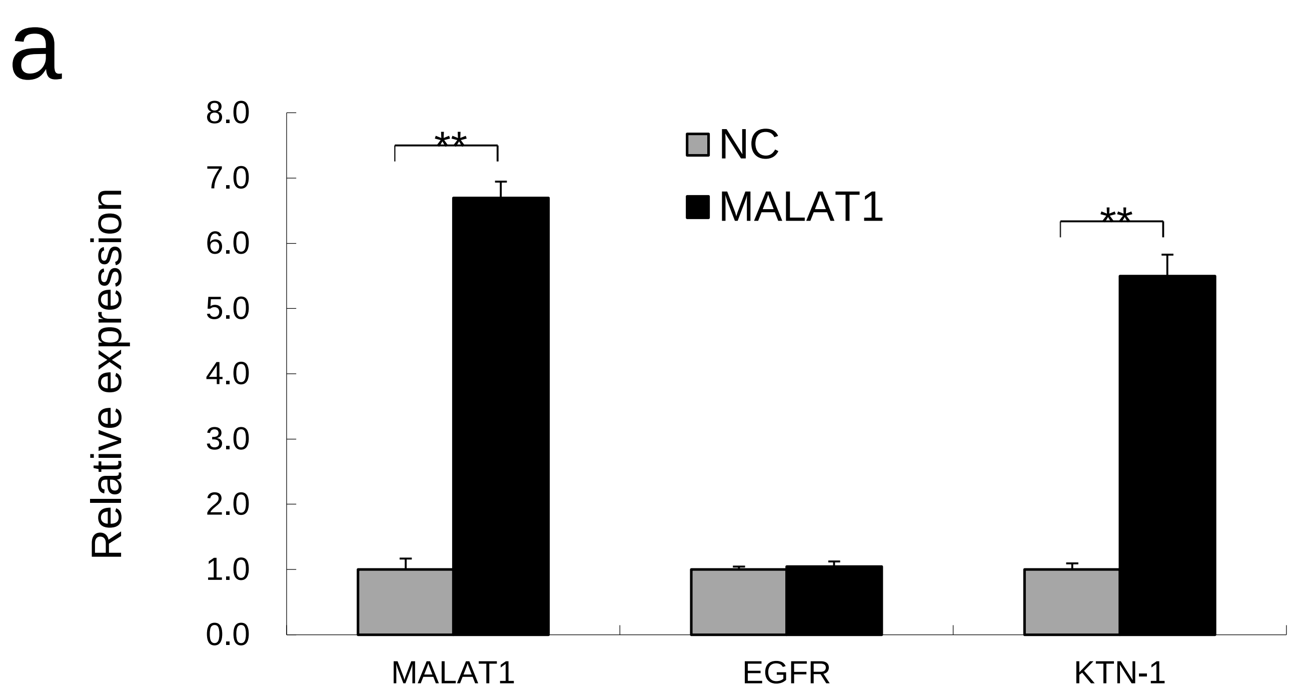

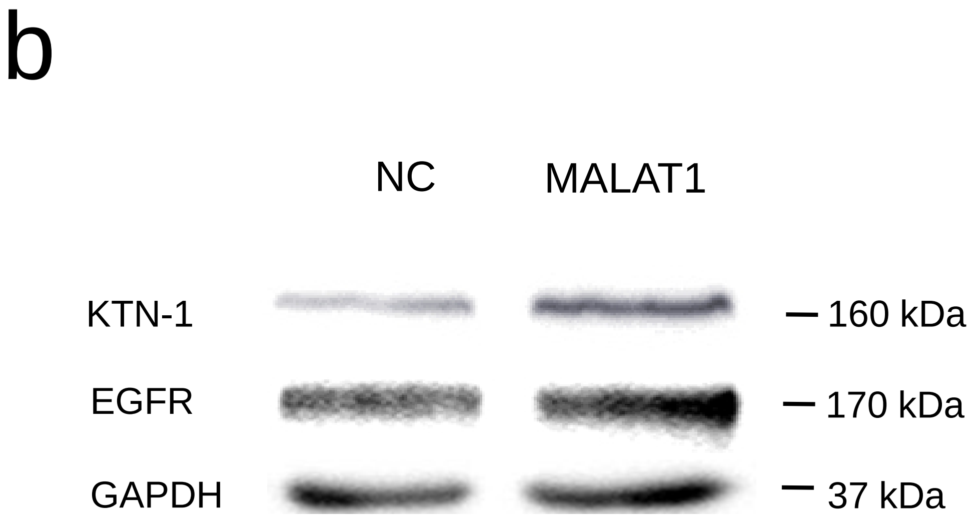

Supplementary Figure S11. KTN1 mediates *MALAT1* regulation of EGFR in A431 cells. (a) RNA expression of *EGFR* and *KTN1* after *MALAT1* overexpression was examined in A431 cells by qRT-PCR. (b) EGFR and KTN1 protein expression was examined after *MALAT1* overexpression in A431 cells by western blot, which showed significant increase of EGFR and KTN1 in response to *MALAT1* overexpression. All statistical data represent the average of three independent experiments ±s.d. * $P < 0.05$, ** $P < 0.01$, *** $P < 0.001$.

Supplementary Figure S12

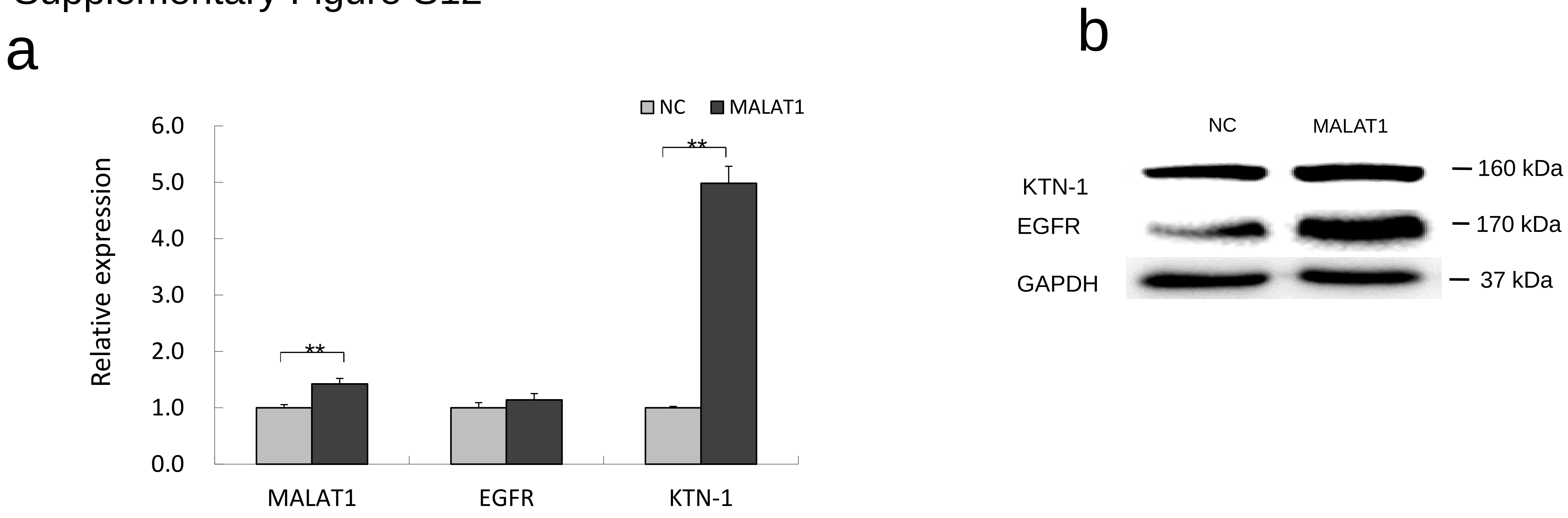

Supplementary Figure S12. KTN1 mediates *MALAT1* regulation of EGFR in HSC-1 cells. (a) RNA expression of *EGFR* and *KTN1* after *MALAT1* overexpression was examined in HSC-1 cells by qRT-PCR. (b) EGFR and KTN1 protein expression was examined after *MALAT1* overexpression in HSC-1 cells by western blot, which showed significant increase of EGFR and KTN1 in response to *MALAT1* overexpression. All statistical data represent the average of three independent experiments ± s.d. * $P < 0.05$, ** $P < 0.01$, *** $P < 0.001$.

Supplementary Figure S13

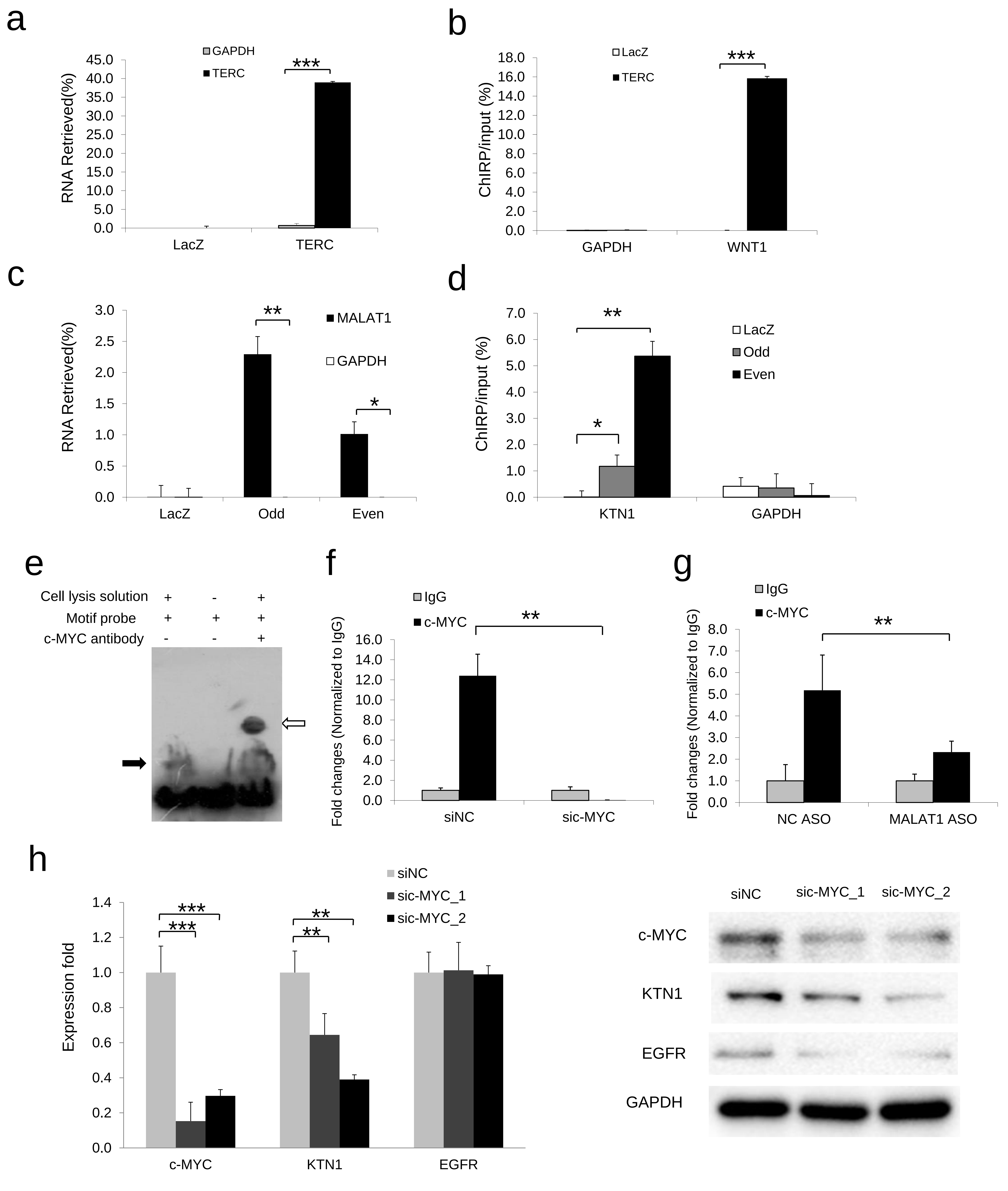

Supplementary Figure S13. The molecular mechanism of *MALAT1* regulation of KTN1 by directly interacting with c-MYC for binding to the promoter region of *KTN1* gene is validated in HSC-1 cell line. (a) Chromatin isolation by RNA purification (ChIRP) with antisense oligos tiling *TERC* showing degree and specificity of *TERC* retrieval. LacZ tiling oligo retrieval of *TERC* RNA was used as a negative control. (b) Relative retrieval of genomic DNAs corresponding to the WNT1 promoter or GAPDH locus following *TERC* or LacZ ChIRP. (c) ChIRP assay with both even and odd antisense oligos tiling *MALAT1* showing degree and specificity of *MALAT1* retrieval. LacZ tiling oligo retrieval of *MALAT1* RNA was used as a negative control. (d) Relative retrieval of genomic DNAs corresponding to the *KTN1* promoter or GAPDH locus following *MALAT1* or LacZ ChIRP. (e) Identification of the E-box element for c-MYC binding at the *KTN1* promoter region. TSS, transcription start site. (f) Electrophoretic mobility shift assay (EMSA) was used to detect the direct association between the E-box element and purified c-MYC protein. Black arrow indicates the binding complex between c-MYC protein and probe containing E-box motif while the white arrow indicates the supershift generated by the association of anti-c-MYC antibody with the above complex. (g) c-MYC siRNA and NC oligos were transfected into HSC-1 cells. The enrichment of c-MYC at the *KTN1* promoter was measured by ChIP-PCR. (f) *MALAT1* and NC ASOs were transfected into HSC-1 cells. The enrichment of c-MYC at the *KTN1* promoter was measured by ChIP-qPCR. (h) RNA interference (RNAi) with siRNAs targeting c-MYC was applied to HSC-1 cells and the protein expression of c-MYC, KTN1, and EGFR was detected using western blot. All statistical data represent the average of three independent experiments ± s.d. * $P < 0.05$, ** $P < 0.01$, *** $P < 0.001$.

Supplementary Figure S14

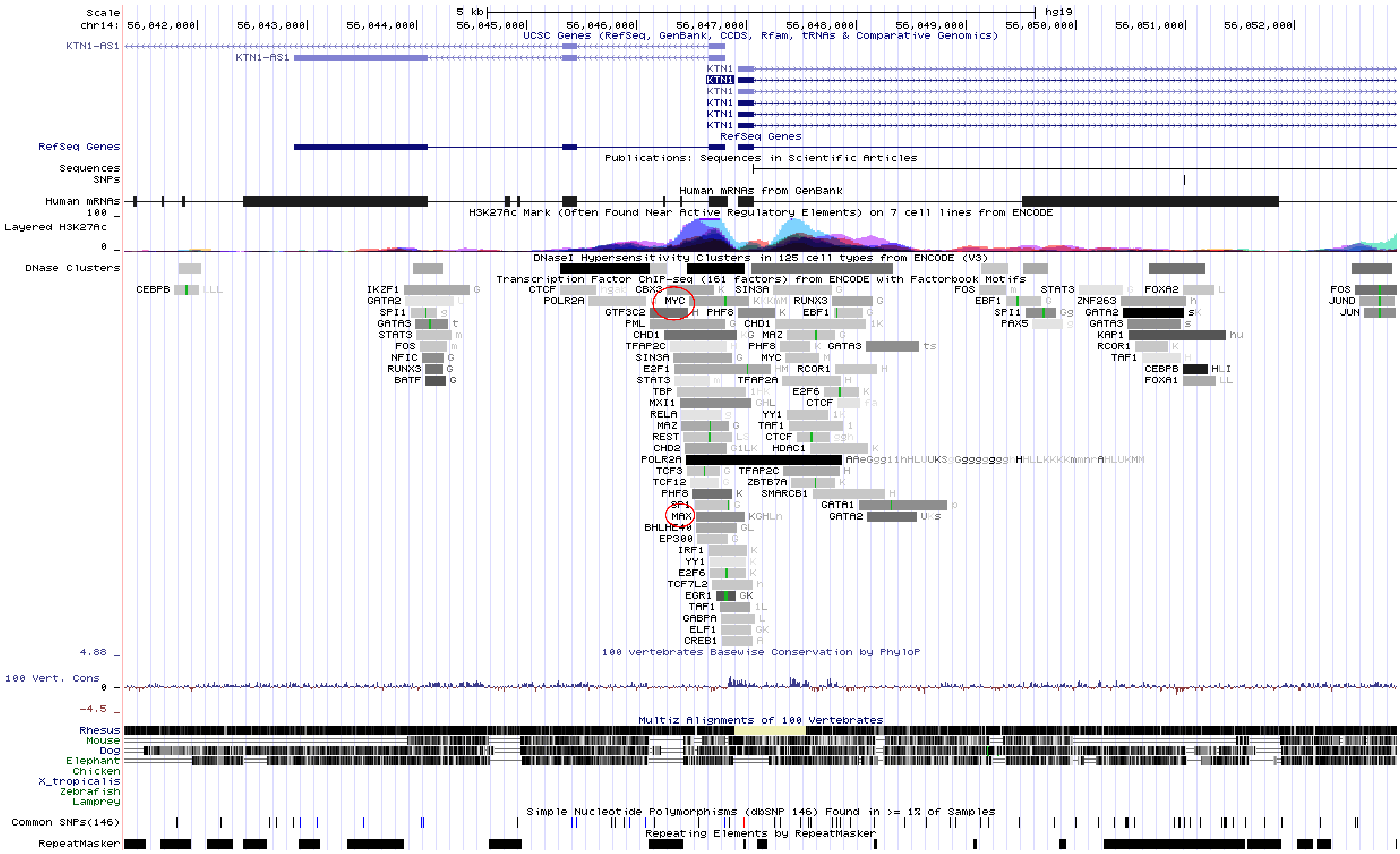

Supplementary Figure S14. Coverage plot showing the ~10 kb genomic region encompassing the transcription start site and promoter region of *KTN1* gene. The red circles labelled out c-MYC and MAX and showed that their coverage regions are mainly overlapped. The coverage regions of transcription factors are provide by Transfac Matrix and Factor databases in UCSC genome browser (https://genome.ucsc.edu/).

Supplementary Figure S15

a

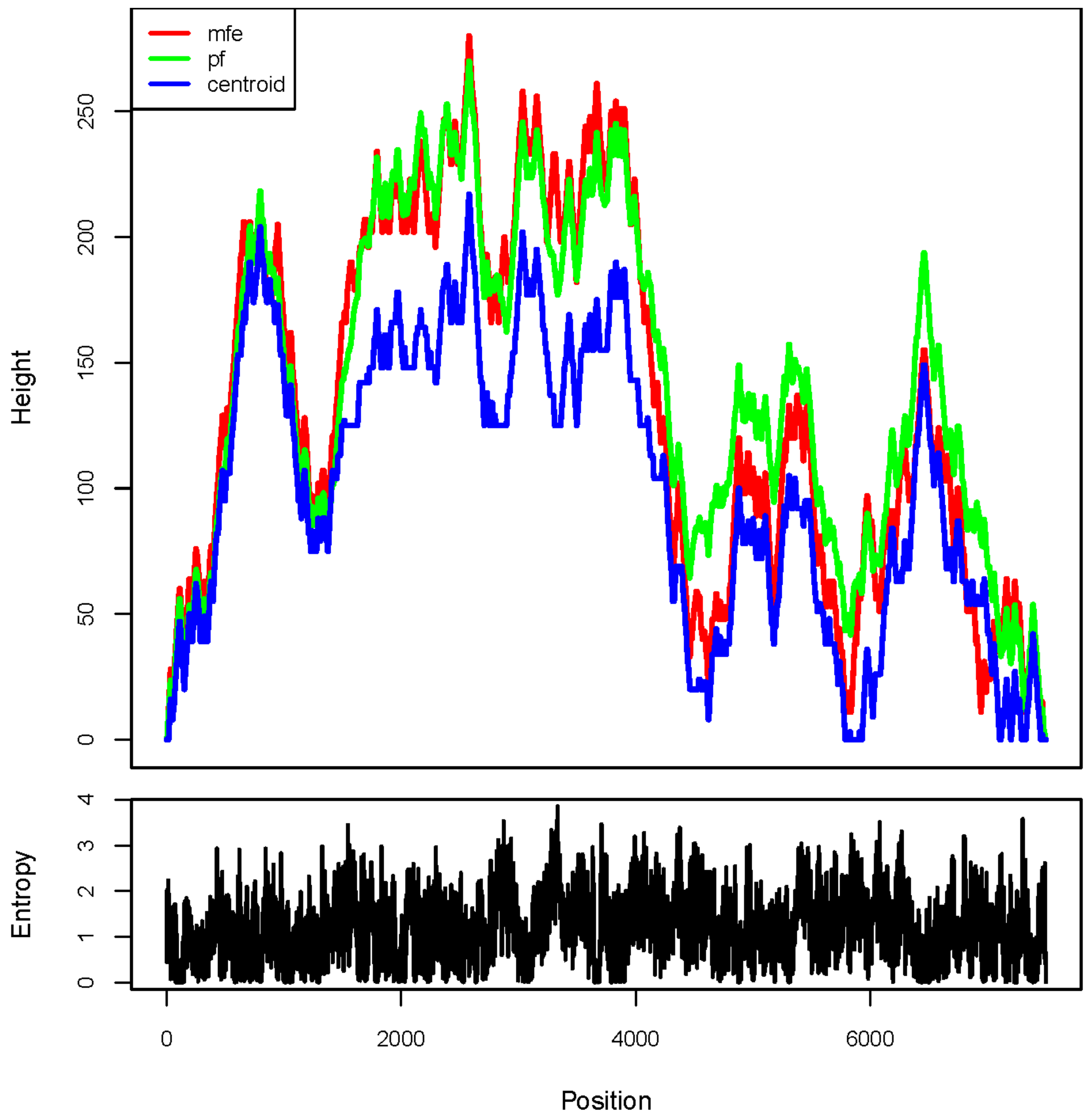

Supplementary Figure S15. Mountain plot representation of the MFE structure of *MALAT1*, the thermodynamic ensemble of RNA structures, and the centroid structure. Additionally the positional entropy for each position is presented at the bottom.

Supplementary Figure S16

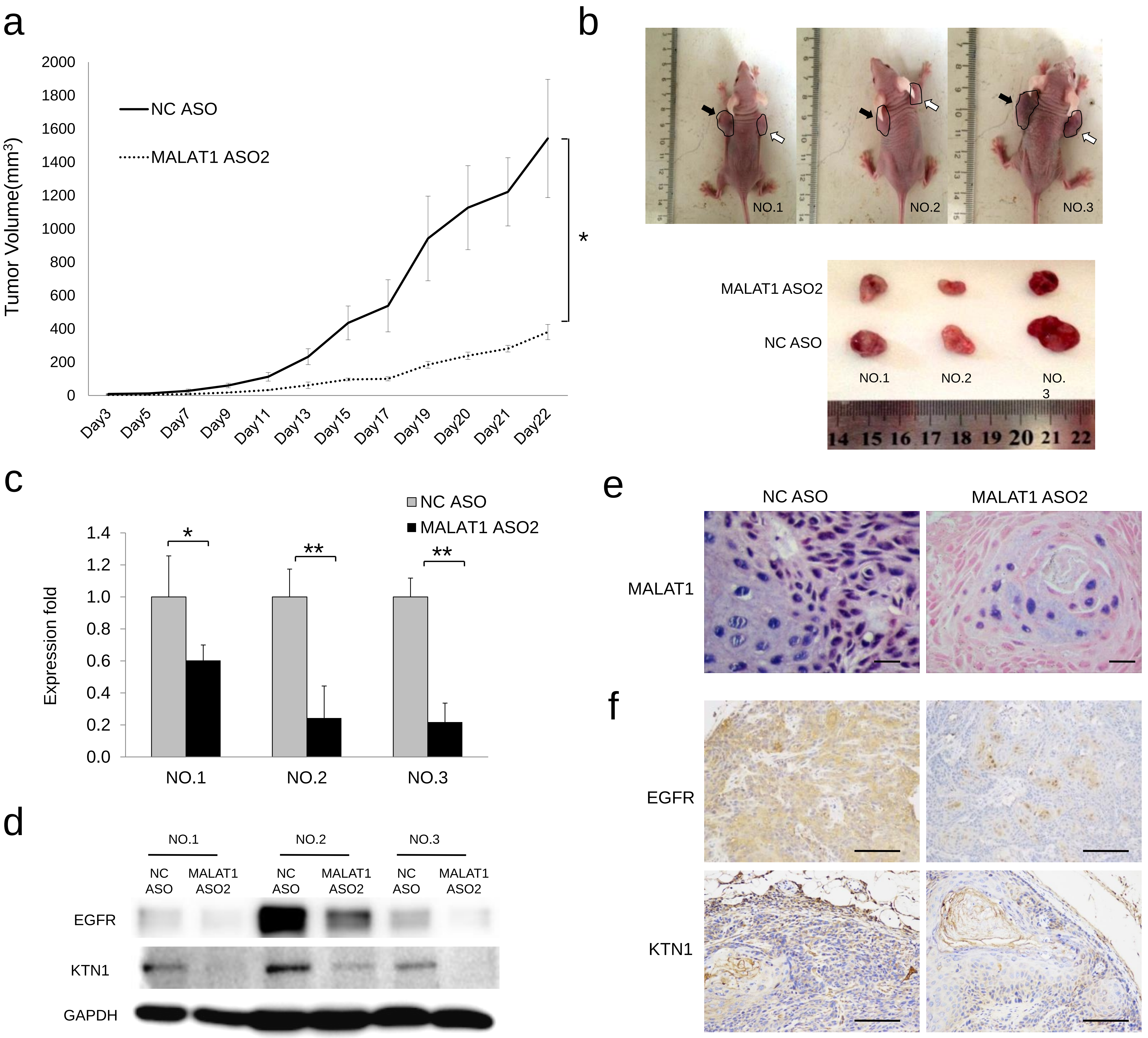

Supplementary Figure S16. The *MALAT1*-KTN1-EGFR regulatory axis exists *in vivo*. *MALAT1* ASO were injected into A431 xenografts every two days. (a) Loss of *MALAT1* attenuates subcutaneous tumor growth in a mouse xenograft model. Tumor volumes (mm^3) were plotted according to days. Tumor volumes statistical data represent the average of three independent experiments ±s.d, respectively. (b) The mice were sacrificed at the end of the experiment and images taken along with the dissected tumors from five representative mice are shown. Black arrows indicate NC ASO-treated xenografts while white arrows indicate *MALAT1* ASO-treated xenografts. (c) The expression of *MALAT1* in the dissected tumors was measured by qRT-PCR. qRT-PCR statistical data represent the average of three independent experiments ±s.d, respectively. (d) The protein expression of KTN1 and EGFR was detected in the xenografts. (e) RNA *in situ* hybridization assay was used to detect expression of *MALAT1* in NC ASO and *MALAT1* ASO-treated xenografts. Scale bar, 50 µm. (f) Histopathology analysis (IHC staining) showing the marked loss of the expression of EGFR and KTN1 in xenografts. Scale bar, 100 µm. *P < 0.05, **P < 0.01, ***P < 0.001.

Supplementary Figure S17

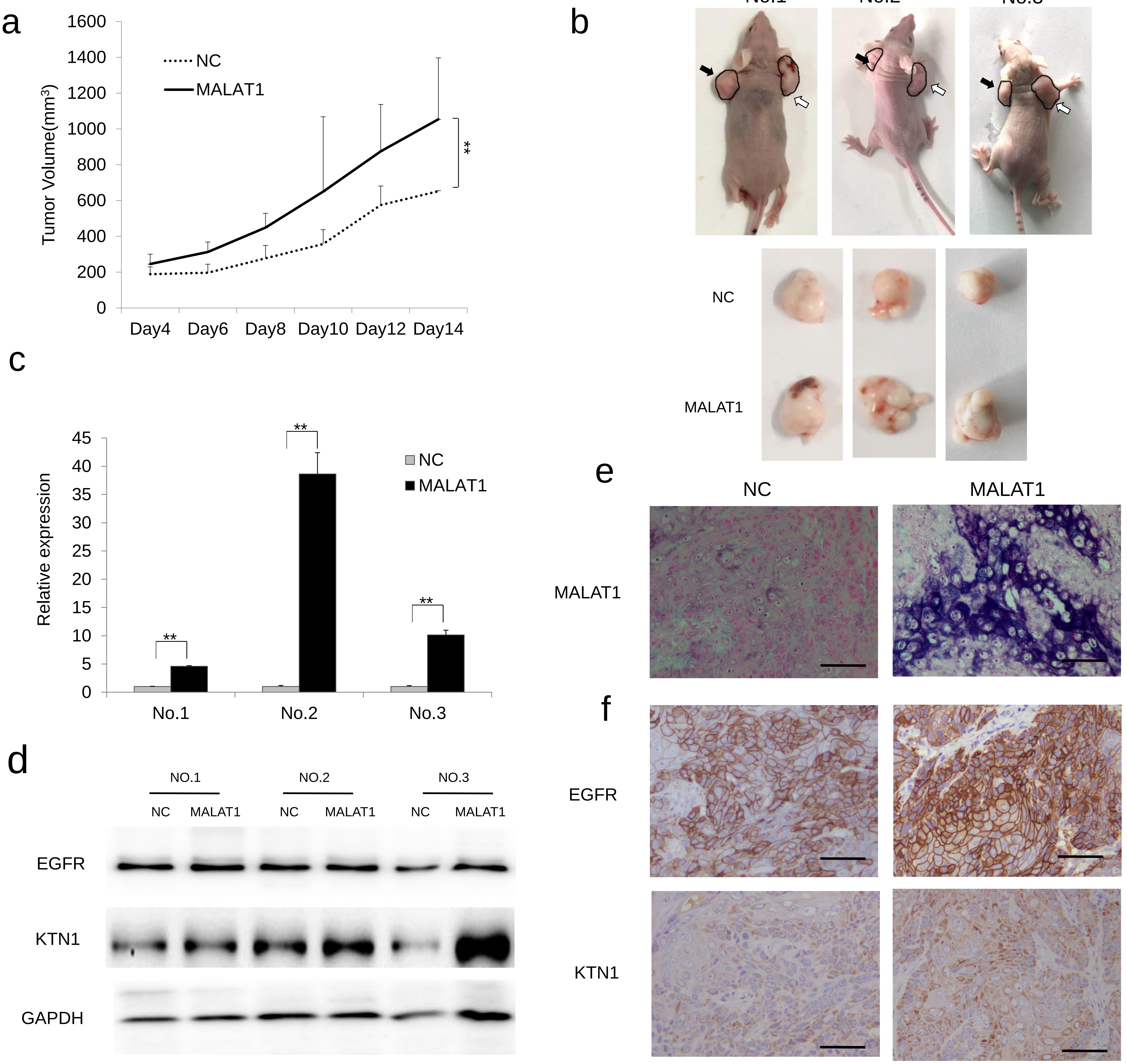

Supplementary Figure S17. The *MALAT1*-KTN1-EGFR regulatory axis exists *in vivo*. *MALAT1* plasmid were injected into A431 cell xenografts every two days. (a) Over expression of *MALAT1* promotes subcutaneous tumor growth in a mouse xenograft model. Tumor volumes (mm^3) were plotted according to days. Tumor volumes statistical data represent the average of three independent experiments ± s.d, respectively. (b) The mice were sacrificed at the end of the experiment and images taken along with the dissected tumors from three representative mice are shown. Black arrows indicate NC-treated xenografts while white arrows indicate *MALAT1*-overexpressed xenografts. (c) The expression of *MALAT1* in the dissected tumors was measured by qRT-PCR. qRT-PCR statistical data represent the average of three independent experiments ±s.d, respectively. (d) The protein expression of KTN1 and EGFR was detected in the xenografts. (e) RNA *in situ* hybridization assay was used to detect expression of *MALAT1* in NC and *MALAT1*-overexpressed xenografts. Scale bar, 50 µm. (f) Histopathology analysis (IHC staining) showing the marked loss of the expression of EGFR and KTN1 in xenografts. Scale bar, 100 µm. *$P < 0.05$, **$P < 0.01$, ***$P < 0.001$.
