# Supplementary material for: MALAT1-KTN1-EGFR regulatory axis promotes the development of cutaneous squamous cell carcinoma
Source: Cell Death Differ. 2019 Jan 25;26(10):2061–73. doi: 10.1038/s41418-019-0288-7 (PMC6748142; doi:10.1038/s41418-019-0288-7)
Supplement: Supplementary file 4 — Supplementary Table 3 [file 41418_2019_288_MOESM4_ESM.pdf]

Supporting Information Table 3: Functional annotation chart of down-regulated genes in MALAT1 depletion cells

Category	Term	Count	%	PValue	Genes	List Total	Pop Hits	Pop Total	Fold Enricl	Bonferroni	Benjamini	FDR
GOTERM_CC_FAT	GO:0012505~endomembrane system	6	3.896104	2.87E-04	HSP90B1, CENPF, KTN1, TPR, B2M, GOLGB1	12	782	12782	8.172634	0.032162	0.032162	0.32559
SP_PIR_KEYWORDS	coiled coil	7	4.545455	3.84E-04	CENPF, KTN1, TPR, DST, ASPM, SMC4, GOLGB1	12	2019	19235	5.557413	0.030287	0.030287	0.407867
SP_PIR_KEYWORDS	phosphoprotein	11	7.142857	4.24E-04	HSP90B1, HNRNPA2B1, CENPF, KTN1, TPR, ITGB1, DST, TOP2A, ASPM, SMC4, GOLGB1	12	7263	19235	2.427658	0.033371	0.016827	0.450021
GOTERM_BP_FAT	GO:0022402~cell cycle process	5	3.246753	7.86E-04	CENPF, ITGB1, DST, ASPM, SMC4	12	565	13528	9.976401	0.217446	0.217446	1.05088
GOTERM_BP_FAT	GO:0007059~chromosome segregation	3	1.948052	0.001881	CENPF, TOP2A, SMC4	12	81	13528	41.75309	0.444162	0.254454	2.498616
GOTERM_BP_FAT	GO:0007049~cell cycle	5	3.246753	0.002562	CENPF, ITGB1, DST, ASPM, SMC4	12	776	13528	7.263746	0.550866	0.234183	3.389983
GOTERM_BP_FAT	GO:0000278~mitotic cell cycle	4	2.597403	0.002844	CENPF, ITGB1, ASPM, SMC4	12	370	13528	12.18739	0.588705	0.199173	3.755632
GOTERM_MF_FAT	GO:0008022~protein C-terminus binding	3	1.948052	0.003142	CENPF, DST, TOP2A	9	141	12983	30.69267	0.164192	0.164192	3.070845
GOTERM_MF_FAT	GO:0046983~protein dimerization activity	4	2.597403	0.003462	CENPF, ITGB1, TOP2A, SMC4	9	542	12983	10.64617	0.17937	0.094114	3.379263
GOTERM_BP_FAT	GO:0022403~cell cycle phase	4	2.597403	0.003909	CENPF, ITGB1, ASPM, SMC4	12	414	13528	10.89211	0.705351	0.216821	5.128809
SP_PIR_KEYWORDS	mitosis	3	1.948052	0.004679	CENPF, ASPM, SMC4	12	183	19235	26.27732	0.312874	0.117573	4.864225
GOTERM_CC_FAT	GO:0005694~chromosome	4	2.597403	0.006157	CENPF, TPR, TOP2A, SMC4	12	460	12782	9.262319	0.50543	0.296743	6.782779
GOTERM_MF_FAT	GO:0046982~protein heterodimerization activity	3	1.948052	0.006712	ITGB1, TOP2A, SMC4	9	208	12983	20.80609	0.318781	0.120109	6.457532
GOTERM_BP_FAT	GO:0045841~negative regulation of mitotic metaphase/anaph	2	1.298701	0.008911	CENPF, TPR	12	11	13528	204.9697	0.938752	0.372161	11.33743
GOTERM_BP_FAT	GO:0007094~mitotic cell cycle spindle assembly checkpoint	2	1.298701	0.008911	CENPF, TPR	12	11	13528	204.9697	0.938752	0.372161	11.33743
SP_PIR_KEYWORDS	cell division	3	1.948052	0.009512	CENPF, ASPM, SMC4	12	264	19235	18.21496	0.534472	0.173988	9.661375
GOTERM_BP_FAT	GO:0031577~spindle checkpoint	2	1.298701	0.009718	CENPF, TPR	12	12	13528	187.8889	0.952491	0.352905	12.30248
GOTERM_BP_FAT	GO:0051784~negative regulation of nuclear division	2	1.298701	0.009718	CENPF, TPR	12	12	13528	187.8889	0.952491	0.352905	12.30248
GOTERM_BP_FAT	GO:0045839~negative regulation of mitosis	2	1.298701	0.009718	CENPF, TPR	12	12	13528	187.8889	0.952491	0.352905	12.30248
GOTERM_CC_FAT	GO:0015630~microtubule cytoskeleton	4	2.597403	0.010043	CENPF, DST, TOP2A, ASPM	12	549	12782	7.760777	0.683569	0.318562	10.84456
SP_PIR_KEYWORDS	acetylation	6	3.896104	0.010827	HNRNPA2B1, CENPF, TPR, ITGB1, TOP2A, SMC4	12	2635	19235	3.649905	0.581405	0.159846	10.92817
KEGG_PATHWAY	hsa05200:Pathways in cancer	3	1.948052	0.011914	HSP90B1, TPR, ITGB1	4	328	5085	11.62729	0.174506	0.174506	8.15939
GOTERM_CC_FAT	GO:0043232~intracellular non-membrane-bounded organelle	7	4.545455	0.012545	HNRNPA2B1, CENPF, TPR, DST, TOP2A, ASPM, SMC4	12	2596	12782	2.872175	0.762889	0.302189	13.37462
GOTERM_CC_FAT	GO:0043228~non-membrane-bounded organelle	7	4.545455	0.012545	HNRNPA2B1, CENPF, TPR, DST, TOP2A, ASPM, SMC4	12	2596	12782	2.872175	0.762889	0.302189	13.37462
GOTERM_BP_FAT	GO:0033043~regulation of organelle organization	3	1.948052	0.012806	CENPF, TPR, DST	12	217	13528	15.58525	0.98207	0.395083	15.90837
GOTERM_BP_FAT	GO:0007067~mitosis	3	1.948052	0.013146	CENPF, ASPM, SMC4	12	220	13528	15.37273	0.983897	0.367926	16.29671
GOTERM_BP_FAT	GO:0000280~nuclear division	3	1.948052	0.013146	CENPF, ASPM, SMC4	12	220	13528	15.37273	0.983897	0.367926	16.29671
GOTERM_BP_FAT	GO:0000087~M phase of mitotic cell cycle	3	1.948052	0.013605	CENPF, ASPM, SMC4	12	224	13528	15.09821	0.986074	0.347797	16.81893
GOTERM_BP_FAT	GO:0048285~organelle fission	3	1.948052	0.014189	CENPF, ASPM, SMC4	12	229	13528	14.76856	0.988424	0.333252	17.47858
UP_SEQ_FEATURE	domain:CH 2	2	1.298701	0.015434	DST, ASPM	12	27	19113	117.9815	0.908859	0.908859	17.02149
UP_SEQ_FEATURE	domain:CH 1	2	1.298701	0.015434	DST, ASPM	12	27	19113	117.9815	0.908859	0.908859	17.02149
INTERPRO	IPR003594:ATP-binding region, ATPase-like	2	1.298701	0.016095	HSP90B1, TOP2A	10	30	16659	111.06	0.56287	0.56287	14.52698
GOTERM_BP_FAT	GO:0030071~regulation of mitotic metaphase/anaphase trans	2	1.298701	0.017751	CENPF, TPR	12	22	13528	102.4848	0.996257	0.37228	21.3972
SMART	SM00387:HATPase_c	2	1.298701	0.019668	HSP90B1, TOP2A	7	30	9079	86.46667	0.242779	0.242779	12.62283
GOTERM_BP_FAT	GO:0030261~chromosome condensation	2	1.298701	0.020149	TOP2A, SMC4	12	25	13528	90.18667	0.998254	0.38646	23.93824
GOTERM_BP_FAT	GO:0010948~negative regulation of cell cycle process	2	1.298701	0.020149	CENPF, TPR	12	25	13528	90.18667	0.998254	0.38646	23.93824
GOTERM_BP_FAT	GO:0051301~cell division	3	1.948052	0.02289	CENPF, ASPM, SMC4	12	295	13528	11.46441	0.999272	0.403128	26.74967
BIOCARTA	h_nkcellsPathway:Ras-Independent pathway in NK cell-mediat	2	1.298701	0.026278	ITGB1, B2M	3	19	1437	50.42105	0.428347	0.428347	18.58555
SP_PIR_KEYWORDS	cell cycle	3	1.948052	0.02732	CENPF, ASPM, SMC4	12	461	19235	10.43113	0.890955	0.308804	25.50829
GOTERM_BP_FAT	GO:0000279~M phase	3	1.948052	0.028055	CENPF, ASPM, SMC4	12	329	13528	10.27964	0.999861	0.446711	31.78636
GOTERM_BP_FAT	GO:0051726~regulation of cell cycle	3	1.948052	0.028372	CENPF, TPR, ITGB1	12	331	13528	10.21752	0.999874	0.429507	32.08528
SP_PIR_KEYWORDS	cytoplasm	6	3.896104	0.028602	HNRNPA2B1, CENPF, DST, TOP2A, ASPM, SMC4	12	3332	19235	2.886405	0.901874	0.282257	26.54546
GOTERM_CC_FAT	GO:0000922~spindle pole	2	1.298701	0.028885	CENPF, ASPM	12	34	12782	62.65686	0.964612	0.487409	28.34707
GOTERM_BP_FAT	GO:0007093~mitotic cell cycle checkpoint	2	1.298701	0.034427	CENPF, TPR	12	43	13528	52.43411	0.999982	0.474267	37.55901
GOTERM_MF_FAT	GO:0000166~nucleotide binding	5	3.246753	0.034681	HSP90B1, HNRNPA2B1, TPR, TOP2A, SMC4	9	2245	12983	3.212819	0.866265	0.39527	29.52167
GOTERM_MF_FAT	GO:0005178~integrin binding	2	1.298701	0.035792	ITGB1, DST	9	59	12983	48.90019	0.874761	0.339994	30.32157
INTERPRO	IPR001715:Calponin-like actin-binding	2	1.298701	0.037197	DST, ASPM	10	70	16659	47.59714	0.855318	0.619629	30.69847
GOTERM_CC_FAT	GO:0044427~chromosomal part	3	1.948052	0.041779	CENPF, TPR, SMC4	12	386	12782	8.278497	0.99229	0.555528	38.45205
GOTERM_BP_FAT	GO:0051783~regulation of nuclear division	2	1.298701	0.04462	CENPF, TPR	12	56	13528	40.2619	0.999999	0.546703	45.86145
GOTERM_BP_FAT	GO:0007088~regulation of mitosis	2	1.298701	0.04462	CENPF, TPR	12	56	13528	40.2619	0.999999	0.546703	45.86145
SMART	SM00033:CH	2	1.298701	0.04539	DST, ASPM	7	70	9079	37.05714	0.478131	0.277595	27.06116
GOTERM_BP_FAT	GO:0051235~maintenance of location	2	1.298701	0.050845	HSP90B1, ASPM	12	64	13528	35.22917	1	0.575524	50.41552
GOTERM_MF_FAT	GO:0005524~ATP binding	4	2.597403	0.053047	HSP90B1, TPR, TOP2A, SMC4	9	1477	12983	3.906718	0.955257	0.404171	41.74087
GOTERM_MF_FAT	GO:0032559~adenyl ribonucleotide binding	4	2.597403	0.054894	HSP90B1, TPR, TOP2A, SMC4	9	1497	12983	3.854524	0.95997	0.368547	42.85775
GOTERM_CC_FAT	GO:0070013~intracellular organelle lumen	5	3.246753	0.054897	HSP90B1, HNRNPA2B1, CENPF, TPR, TOP2A	12	1779	12782	2.993723	0.998398	0.601289	47.38231
GOTERM_BP_FAT	GO:0007229~integrin-mediated signaling pathway	2	1.298701	0.055489	ITGB1, DST	12	70	13528	32.20952	1	0.589579	53.57946
GOTERM_MF_FAT	GO:0042802~identical protein binding	3	1.948052	0.05575	CENPF, ITGB1, TOP2A	9	640	12983	6.761979	0.961984	0.335498	43.36839
GOTERM_BP_FAT	GO:0051276~chromosome organization	3	1.948052	0.05695	CENPF, TOP2A, SMC4	12	485	13528	6.973196	1	0.581536	54.53557
SP_PIR_KEYWORDS	heterodimer	2	1.298701	0.057366	ITGB1, B2M	12	103	19235	31.1246	0.991139	0.446098	46.63709
GOTERM_CC_FAT	GO:0043233~organelle lumen	5	3.246753	0.05898	HSP90B1, HNRNPA2B1, CENPF, TPR, TOP2A	12	1820	12782	2.926282	0.999022	0.579483	49.91019
GOTERM_CC_FAT	GO:0031090~organelle membrane	4	2.597403	0.061597	HSP90B1, KTN1, B2M, GOLGB1	12	1096	12782	3.88747	0.999288	0.55304	51.4715
GOTERM_MF_FAT	GO:0030554~adenyl nucleotide binding	4	2.597403	0.062619	HSP90B1, TPR, TOP2A, SMC4	9	1577	12983	3.658987	0.974926	0.33605	47.32231
GOTERM_CC_FAT	GO:0031974~membrane-enclosed lumen	5	3.246753	0.062707	HSP90B1, HNRNPA2B1, CENPF, TPR, TOP2A	12	1856	12782	2.869522	0.999378	0.522055	52.12075

GOTERM_BP_FAT	GO:0045786~negative regulation of cell cycle	2	1.298701	0.06395	CENPF, TPR	12	81	13528	27.83539	1	0.608284	58.86813
GOTERM_CC_FAT	GO:0000776~kinetochore	2	1.298701	0.064329	CENPF, TPR	12	77	12782	27.66667	0.999489	0.497969	53.05457
GOTERM_BP_FAT	GO:0010639~negative regulation of organelle organization	2	1.298701	0.064715	CENPF, TPR	12	82	13528	27.49593	1	0.596497	59.31815
GOTERM_MF_FAT	GO:0001883~purine nucleoside binding	4	2.597403	0.06504	HSP90B1, TPR, TOP2A, SMC4	9	1601	12983	3.604136	0.978363	0.318416	48.65567
GOTERM_MF_FAT	GO:0001882~nucleoside binding	4	2.597403	0.066166	HSP90B1, TPR, TOP2A, SMC4	9	1612	12983	3.579542	0.979799	0.298638	49.26516
GOTERM_BP_FAT	GO:0000075~cell cycle checkpoint	2	1.298701	0.071581	CENPF, TPR	12	91	13528	24.77656	1	0.619222	63.15446
GOTERM_BP_FAT	GO:0051640~organelle localization	2	1.298701	0.072341	CENPF, ASPM	12	92	13528	24.50725	1	0.608253	63.55788
GOTERM_CC_FAT	GO:0042470~melanosome	2	1.298701	0.074008	HSP90B1, ITGB1	12	89	12782	23.93633	0.999844	0.518311	58.29061
GOTERM_CC_FAT	GO:0048770~pigment granule	2	1.298701	0.074008	HSP90B1, ITGB1	12	89	12782	23.93633	0.999844	0.518311	58.29061
SP_PIR_KEYWORDS	nucleus	6	3.896104	0.0755	HNRNPA2B1, CENPF, TPR, TOP2A, ASPM, SMC4	12	4283	19235	2.245505	0.998127	0.50232	56.59395
GOTERM_BP_FAT	GO:0050658~RNA transport	2	1.298701	0.076133	HNRNPA2B1, TPR	12	97	13528	23.24399	1	0.613353	65.51011
GOTERM_BP_FAT	GO:0050657~nucleic acid transport	2	1.298701	0.076133	HNRNPA2B1, TPR	12	97	13528	23.24399	1	0.613353	65.51011
GOTERM_BP_FAT	GO:0051236~establishment of RNA localization	2	1.298701	0.076133	HNRNPA2B1, TPR	12	97	13528	23.24399	1	0.613353	65.51011
GOTERM_BP_FAT	GO:0006403~RNA localization	2	1.298701	0.078401	HNRNPA2B1, TPR	12	100	13528	22.54667	1	0.610719	66.63121
GOTERM_CC_FAT	GO:0016023~cytoplasmic membrane-bounded vesicle	3	1.948052	0.078607	HSP90B1, ITGB1, DST	12	550	12782	5.81	0.999912	0.512236	60.58643
GOTERM_BP_FAT	GO:0051329~interphase of mitotic cell cycle	2	1.298701	0.080664	CENPF, ITGB1	12	103	13528	21.88997	1	0.60826	67.71611
GOTERM_BP_FAT	GO:0051325~interphase	2	1.298701	0.082922	CENPF, ITGB1	12	106	13528	21.27044	1	0.605959	68.76597
GOTERM_CC_FAT	GO:0031988~membrane-bounded vesicle	3	1.948052	0.083137	HSP90B1, ITGB1, DST	12	568	12782	5.62588	0.99995	0.506769	62.73494
GOTERM_BP_FAT	GO:0015931~nucleobase, nucleoside, nucleotide and nucleic a	2	1.298701	0.088172	HNRNPA2B1, TPR	12	113	13528	19.9528	1	0.617091	71.08561
GOTERM_BP_FAT	GO:0007018~microtubule-based movement	2	1.298701	0.088172	KTN1, DST	12	113	13528	19.9528	1	0.617091	71.08561
GOTERM_MF_FAT	GO:0003682~chromatin binding	2	1.298701	0.088799	CENPF, TOP2A	9	150	12983	19.23407	0.995011	0.357065	60.21795
GOTERM_BP_FAT	GO:0010564~regulation of cell cycle process	2	1.298701	0.088919	CENPF, TPR	12	114	13528	19.77778	1	0.608296	71.40271
GOTERM_BP_FAT	GO:0006323~DNA packaging	2	1.298701	0.091159	TOP2A, SMC4	12	117	13528	19.27066	1	0.606218	72.33343
GOTERM_MF_FAT	GO:0032555~purine ribonucleotide binding	4	2.597403	0.091221	HSP90B1, TPR, TOP2A, SMC4	9	1836	12983	3.142823	0.995713	0.342562	61.25392
GOTERM_MF_FAT	GO:0032553~ribonucleotide binding	4	2.597403	0.091221	HSP90B1, TPR, TOP2A, SMC4	9	1836	12983	3.142823	0.995713	0.342562	61.25392
GOTERM_CC_FAT	GO:0030055~cell-substrate junction	2	1.298701	0.092306	ITGB1, DST	12	112	12782	19.02083	0.999984	0.520997	66.76017
